# The geodynamic origin of Los Humeros volcanic field in Mexico: insights from numerical simulations

**DOI:** 10.1038/s41598-023-49292-x

**Published:** 2023-12-14

**Authors:** A. Bayona, V. C. Manea, M. Manea, S. Yoshioka, E. Moreno, N. Suenaga

**Affiliations:** 1 Computational Geodynamics Laboratory, Centro de Geociencias, Universidad Nacional Autónoma de México, Campus Juriquilla, 76230 Querétaro, Mexico; 2 Research Center for Urban Safety and Security, Kobe University, Kobe, 657-8501 Japan; 3 Department of Planetology, Graduate School of Science, Kobe University, Kobe, 657-8501 Japan

**Keywords:** Geodynamics, Geophysics

## Abstract

Compared to normal arc-related volcanic eruptions, the formation of a volcanic caldera is a relatively atypical event. During caldera formation a series of large volumes of magma are erupted, reducing the structural support for the rock above the magma chamber and creating a large depression at the surface called caldera. Los Humeros volcanic field (LHVF) represents one of the largest volcanic calderas in Mexico. It is located some 400 km from the trench at the eastern edge of the Trans Mexican Volcanic Belt where the depth to the Cocos slab is more than 300 km. In this study we employ high-resolution two-dimensional thermomechanical numerical simulations of magma intrusions and a horizontal tectonic strain rate to better understand the influence of crustal deformation for the formation of Los Humeros caldera. A minimum number of three thermal anomaly pulses of hydrated mantle material (with diameter of 15 km or more) and a regional strain rate of 7.927 × 10^–16^ s^−1^ are required for magma to reach the surface. Modeling results show that regional extension coupled with deep thermal anomalies (with a temperature excess of ΔT ≥ 100 °C) that come in a specific chain-type sequence produce surface deformation patterns similar to LHVF. We propose an asthenospheric sub-slab deep source (> 300 km depth) for the thermal anomalies where previous studies showed the existence of a gap or tear in the Cocos slab.

## Introduction

**Figure 1:**
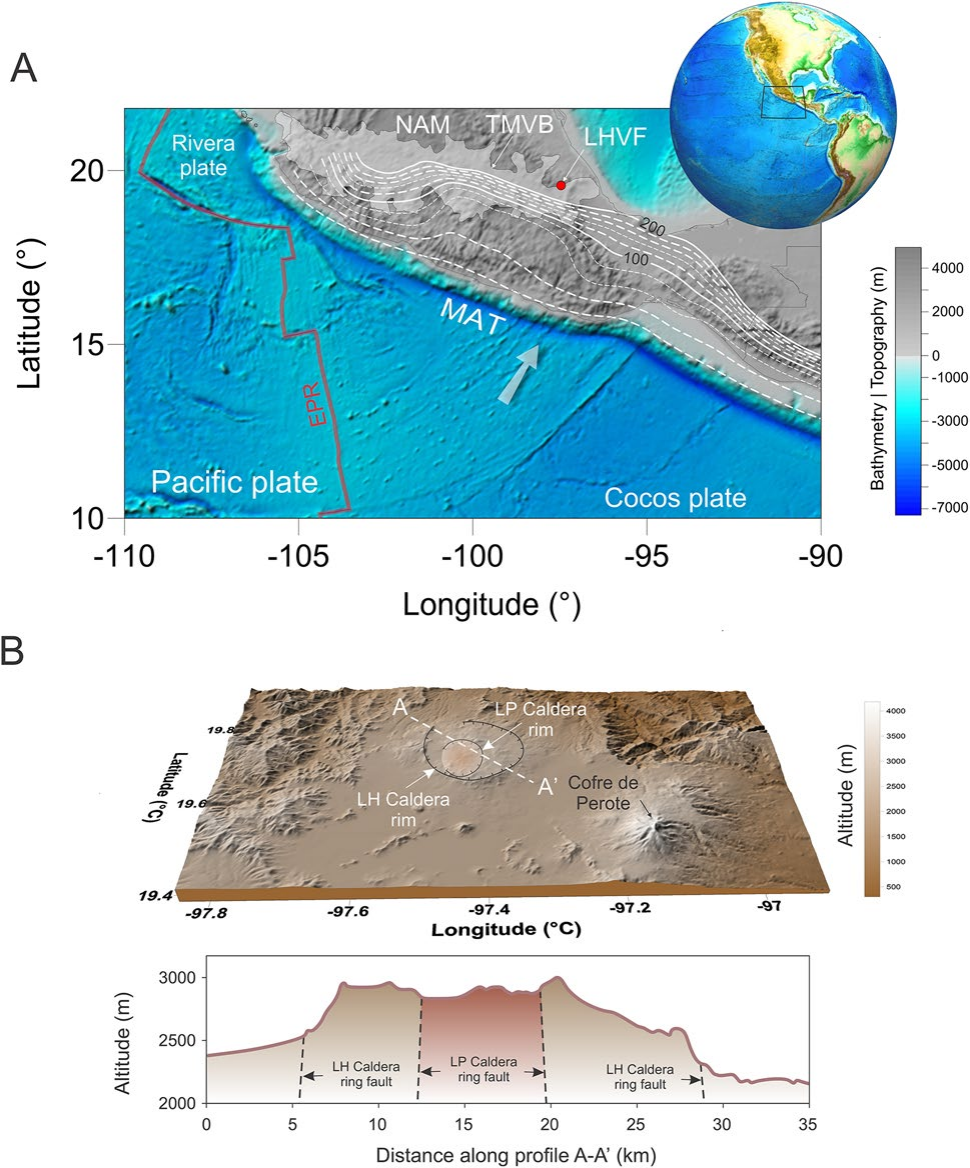
(**a**) Topographic and bathymetric map of the Mexican subduction zone with the location of the LHVF at the eastern edge of the TMVB marked as the red dot. White contours represent the slab depths from SLAB 2.0 model^11^ where thin dashed curves mark slab contours every 20 km. Other notations are: NAM—North America Plate, MAT—Middle America Trench, EPR—East Pacific Rise. TMVB—Trans Mexican Volcanic Belt. White arrow shows the convergence between the Cocos plate and NAM. Upper right inset is generated based on ETOPO1 Global Relief Model dataset^12^. (**b**) LHVF topography showing the location of main Los Humeros (LH) Caldera and Los Potreros (LP) Caldera rims and ring faults. This figure is created using the open-source software ParaView v.5.6.0. (http://www.paraview.org) and Corel Draw 2022.

The location of volcanic fields is strongly related to various tectonic environments such as subduction zones, rifts, and mantle plumes^1–3^. Regional tectonic stresses also play a key role in the localization of volcanic fields. Globally, analysis of intermediate depth earthquakes reveals that the stress field in the arc region is somewhat controlled by the asthenospheric flows^4^. Other parameters as thickness and thermal state of the crust and the lithospheric mantle can also facilitate the migration towards the surface of deep thermal anomalies. Whether the location and origin of volcanic arcs is reasonably well understood^1,5^, origin of volcanic calderas as places of past massive volcanic eruptions in areas distant from subduction zones, such as Los Humeros, remains still elusive. In this context, Mexico is the place of no less than six large volcanic calderas^6^ located along the Trans Mexican Volcanic Belt (TMVB), among which the LHVF is the largest one, and probable one of the best studied^7^. Its unusual location at the eastern edge of the TMVB (Fig. 1), where the depth to the Cocos slab is more than 300 km and distance to the Middle America Trench (MAT) is over 400 km, makes difficult to understand its deep geodynamic origin and possible relationship with the subduction system. TMVB is a geological province between the Pacific coast and the Gulf of Mexico with a length of roughly 1000 km (Fig. 1A), and tectonically controlled by the subduction of the Rivera and Cocos plates beneath the North American plate^8^. The Cocos plate subducts horizontally for 250 km beneath central Mexico to the vicinity of Mexico City, where it sharply bends and subducts with a much steeper angle. Tectonic models propose that at 7 Ma the slab bending edge was actually located closer to the rear of the TMVB, and start to roll back to its current position, inducing crustal extension in the volcanic belt^8^. This regional extension field has an orientation almost perpendicular to the MAT, and exerts a strong structural control in the location of the monogenetic and polygenetic volcanoes along the TMVB^9^. Additionally, several studies propose that the Cocos slab is affected by a trench parallel tear that propagated from the Gulf of California^8,10^. Despite the abundance of tectonic related studies with the Mexican subduction zone, the asthenospheric origin of magma source associated with LHVF is not yet well understood and investigated.

In this study we present two-dimensional high-resolution thermomechanical numerical models of magma intrusion for LHVF. We decided to investigate this region because it holds some key elements for better understanding its origin, as the largest caldera in the TMVB^13,14^, and its unusual location (> 400 km) from the MAT where; the Cocos slab is about 300 km deep. This study specifically investigates the conditions which allow deeply located magmatic sources to reach surface and quantify their superficial effects as surface deformation patterns. Additionally, we investigate the influence of evolution of stress conditions in the crust on the LHVF formation. Modeling results show that best models that produce surface deformation patters similar to LHVF caldera are obtained when a series of thermal anomalies (ΔT ≥ 100 °C) that migrate towards the surface in a specific chain-type sequence is coupled with regional crustal extension. Given the isotopic signature^15–17^ and the specific tectonic setting of the area where LHVF is located^9,18,19^, the rear part of the TMVB, which is characterized by alkaline mafic volcanism related to a slab detachment event^8,10,20^, we propose a sub slab asthenospheric origin for the thermal anomalies that created this large volcanic caldera in Mexico.

## Los Humeros volcanic field

LHVF was formed by two main collapse events and the emplacement of several monogenetic volcanoes within and along the caldera’s border^21^, and Los Humeros is the largest and the oldest caldera (0.164 Ma). It is composed of four stratigraphic units, basement, pre-calderic phase, calderic phase and post-calderic phase, which are separated by unconformities that mark different geological events showing their evolution. The basement includes all the igneous, metamorphic and sedimentary rocks older than 1.44 Ma^22^, the pre-calderic phase encompasses the rhyolitic domes that overlie the basement and that were formed prior to the calderic events aged 0.69–0.27 Ma^23^, the calderic phase ranges from the Xaltipan Ignimbrite (Los Humeros (LH) caldera formation) to the Zaragoza Ignimbrite (Los Potreros (LP) caldera formation) aged 0.149–0.069 Ma^23^, and finally the post-calderic phase is made up of magmatism from the last 0.05 Ma^23^ associated with minor eruptions, lava flows, and also scoria cones^13,14,24^ (Fig. 1B). Seismological studies have shown horizontal accumulation of hydrated magmas under the TMVB (reaching the rear of the arc) near the crust-mantle boundary^25^, and recent geochemical studies have found a signature of slab-derived components in the calderic and post-calderic units of Los Humeros^26^, which might indicate that the magmas are linked to the Cocos slab. Lately, studies related to the post-calderic phase and current activity in LHVF have been carried out, such as studies of analogous and hydrothermal models^27,28^, evolution of intrusions^29^, seismic images^30^, and description of a new system of small pockets instead of large magma chamber within the crust^31^, among others, which explain the configuration and current activity of the field. Although the potential asthenospheric origin of LHVF is still not understood, several lines of evidence generate a series of hypotheses regarding its formation. Our work focuses on numerical models of the deep geodynamic origin that gave rise to LHVF. The current magmatism in the rear zone of the TMVB has been associated with crustal extension since the Upper Miocene^25,32^. Mesozoic sedimentary rocks and Precambrian-Paleozoic metamorphic rocks of the LHVF basement experimented a compressional orogenic phase by the Mexican Fold and Thrust Belt (MFTB) in the Upper Cretaceous-Eocene^33^, which generated thrust faulting and folding^34^. Besides, the pre-existing volcanoes in the region modified the local stress field, inducing deformation in the basement, generating faults with different geometries and kinematics^35^. In this region, the MFTB reached 4–5 km depth^35^. This compression had its greatest stress component in the northeast-southwest direction^35^. After this compressional phase, LHVF underwent extensional tectonic deformation in the Eocene-Pliocene associated with scattered northeast trending normal faults, which facilitated the magmatic intrusions of the Eocene–Oligocene, preceding the beginning of the volcanism of the TMVB. An extensional tectonic phase has occurred in the LHVF since the Miocene, which is contemporaneous with the emplacement of the magmas in the arc. The structural analyses suggest that the deformation of the basement and the compressional-extensional events played an important role for the formation of the LHVF and the postcalderic activity^27,35^. It is widely accepted that under certain stress conditions, the regime facilitates the decompression of the upper mantle, making it possible for asthenospheric magmas to rise. This is true, nonetheless, some sediment-related geochemical signature of the slab indicates it might not be the mechanism. Furthermore, the small extension rate is insufficient to generate magmas by decompression in the upper mantle that can reach the surface^36^. Another hypothesis, such as the existence of mantle plumes, is not validated by geochemical and geophysical information^26^. Here we propose that rollback of the Cocos slab caused extension in the TMVB^8^, and at the same time above slab mantle wedge hydrated buoyant pockets were entrained by the mantle flow at higher depths from where they migrate back and reach the base of the lithosphere beneath the LHVF^37^. Based on this hypothesis, we propose to advance our understanding of LHVF formation and use high-resolution two-dimensional thermomechanical numerical simulations of magma intrusion tailored to the above mentioned calderic volcanic events recorded at LHVF.

## Modeling results

**Figure 2:**
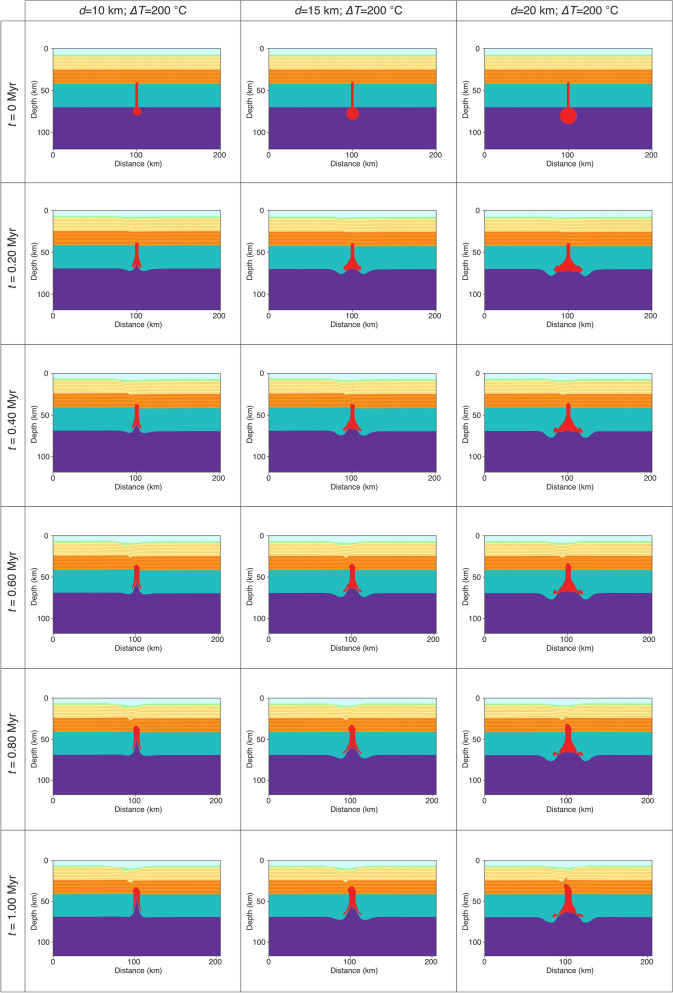
Time series showing modeling results with a single thermal anomaly of 10, 15 and 20 km in diameter and a *ΔT* = 200 °C. All the numerical simulations are performed with an extensional background strain rate of 7.927 × 10^–16^ s^−1^ (or ± 2.5 mm/yr applied on each side of the model domain) and integrated 1 Myr in time.

We developed high-resolution two-dimensional thermomechanical numerical simulations of mantle hydrated magma intrusions into the continental crust to predict temperature, viscosity, strain rate, surface deformation, and partial melt distribution in the crust beneath LHVF. Governing equations, phase equations and model setup are presented in the Supplementary Information^38^. Numerical simulations include crustal extension rates, sublithospheric thermal anomalies involve a uniform continental crust and asthenosphere with specific model parameters (Fig. S1). Thermal anomalies are time-based inserted into the model at stages tailored on the calderic events specific for LHVF. Our approach towards evaluating the crustal impact of thermal anomalies include a parameter space based on tectonic extension rates, total number of thermal pulses and their initial size (i.e., diameter *d*), and temperature excess (the difference between the thermal anomaly and the surrounding mantle *ΔT*). We investigate the effects of a thermal anomaly temperature in the range of 100–200 °C and anomaly diameter in the range of 10 to 20 km. The initial model setup contains a uniformly stratified crust, lithosphere and asthenosphere, and the thermal anomalies are homogeneous and have spherical shape. To better quantify the effect of thermal anomalies of different sizes and temperature excess, the first set of numerical models does not consider regional tectonic extension and includes both hydrated and dry mantle thermal anomalies. These models with thermal anomalies of 20 km in diameter or less (i.e., 15 km and 10 km) and temperature excess of 200 °C or less, show that the main deformation is concentrated at the base of lithosphere and produce a symmetric lithospheric delamination pattern (Fig. S2). However, the overlying continental crust is not efficiently affected (i.e., formation of major shear bands) as no significant internal deformation is observed. Including unrealistically larger and hotter thermal anomalies (i.e., *d* > 20 km and *ΔT* > 200 °C) also show the same behavior, their energy is mainly consumed for lithospheric delamination processes (Fig. S3). We also performed a series of simulations without crustal extension but this time the thermal anomalies are composed of hydrated mantle. Modeling results (Fig. S4, S5) are similar to the previous ones where only continental lithosphere deformation is observed without crustal effects that eventually allow magma to reach the surface. Since LHVF is characterized by several eruption episodes^23,26^, we also perform models (without crustal extension) with multiple identical thermal anomalies but introduced in the model at difference time instances. However, modeling results show a similar development where only growing in time lithospheric delamination is observed (Fig. S6 and S7). These models show that thermal anomalies (composed of dry or hydrated mantle alike) alone cannot explain the formation of LHVF, and additional factors should also be considered. Since several lines of evidence show that the TMVB is affected by a small degree (max. ~ 3%) of crustal extension^9,18^, our modeling strategy also includes a series of simulations that integrate horizontal extension to the lateral edges of the 200 km wide model domain at strain rates of 1.585–7.927 × 10^–16^ s^−1^ (or ± 0.5–2.5 mm/yr) and are integrated in time 1 Myr. The first set of models of this kind includes only a single initial thermal anomaly, and subsequent simulations include several successive episodes to hydrated mantle thermal anomalies. In Fig. 2 we show a time series of the modeling results for a setup with a single hydrated initial thermal anomaly (*d* = 10–20 km and *ΔT* = 200 °C) and with an extensional strain rate of 7.927 × 10^–16^ s^−1^ (or ± 2.5 mm/yr applied on each side of the model domain). A small degree of lithosphere delamination is observed that is proportional with to the size of the initial thermal anomaly.

These simulations also produce a series of shear bands at crustal level that can be considered the equivalent of normal faults. However, independent of their size and temperature contrast, the thermal anomalies are not able to ascent completely to the surface and remained embedded into the base of the lower crust (Fig. 2). Because these models failed to allow partially molten mantle material to penetrate the entire continental crust, we perform another series of simulations where a secondary thermal anomaly of the same size and diameter is introduced at a certain time step during the model evolution. The main motivation behind this scenario is the LHVF multiple calderic phases (precalderic, sincalderic and postcalderic) aged from 0.69 Ma to 0.05 Ma^23^.

**Figure 3:**
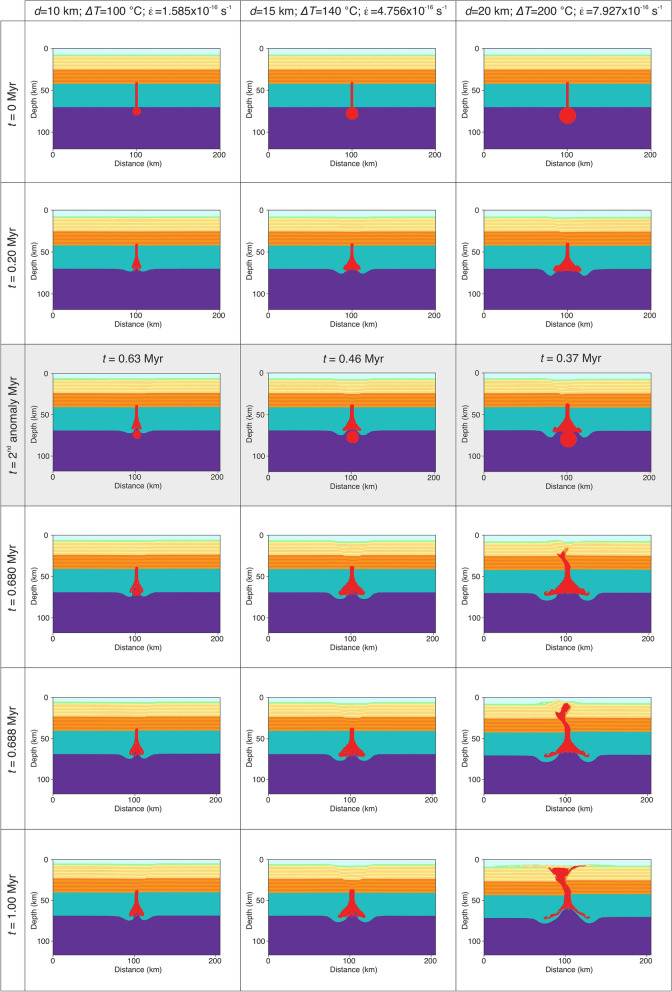
Time series showing modeling results with dual thermal anomalies of 10, 15 and 20 km in diameter and a *ΔT* = 100 °C, 140 °C, and 200 °C and extensional background strain rates of 1.585–4.756–7.927 × 10^–16^ s^−1^. Time snapshots marked with a gray background depict the moment of the secondary thermal anomaly addition. The secondary thermal anomaly has the same properties as the initially set up. All models are integrated 1 Myr in time.

**Figure 4:**
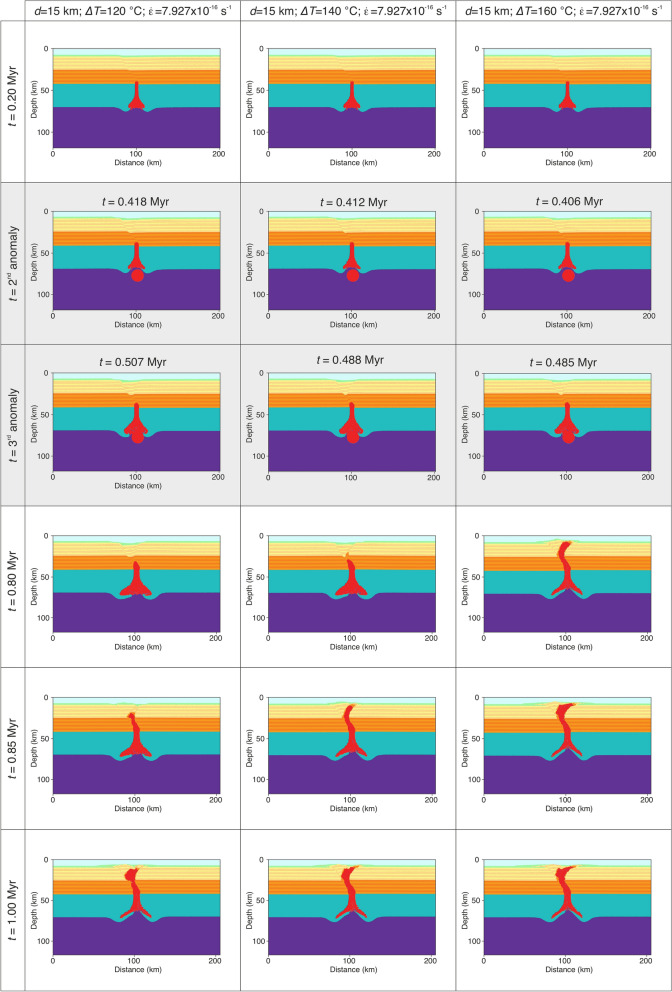
Time series showing modeling results with a series of three thermal anomalies of 15 km in diameter, a *ΔT* = 120 °C, 140 °C, and 160 °C and constant extensional background strain rates of 7.927 × 10^–16^ s^−1^. Time snapshots marked with a gray background depict the moment of the secondary and tertiary thermal anomaly additions. All thermal anomalies have the same properties as initially setup; all models are integrated 1 Myr in time.

**Figure 5:**
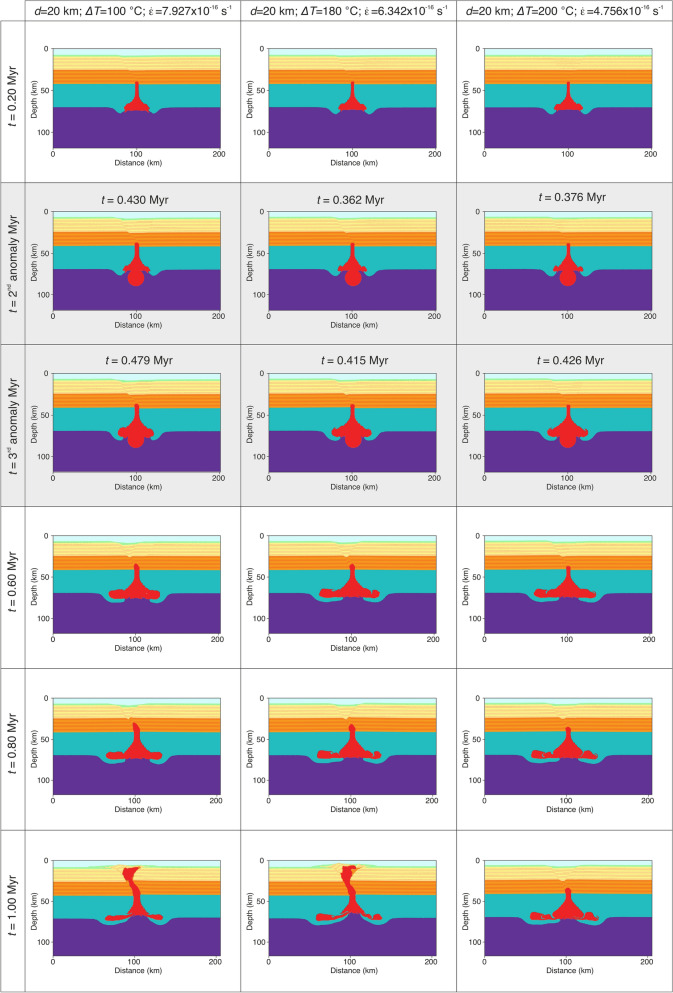
Time series showing modeling results with a series of three thermal anomalies of 20 km in diameter, a *ΔT* = 100 °C, 180 °C, and 200 °C and extensional background strain rates of 7.927–6.342–4.756 × 10^–16^ s^−1^. Time snapshots marked with a gray background depict the moment of the secondary and tertiary thermal anomaly additions. All thermal anomalies have the same properties as initially setup; all models are integrated 1 Myr in time.

For numerical convenience, the secondary thermal plume is inserted after 1000 numerical time steps in all simulations. This corresponds to a geological time of 0.35 Myr to 0.45 Myr after the initial of the simulation, depending on the parameters used. As in the previous models, we varied the anomaly diameter from 10 to 20 km, the thermal anomaly from 100 °C to 200 °C and the extensional strain rate from 1.585 × 10^–16^ s^−1^ to 7.927 × 10^–16^ s^−1^. Modeling results show a similar behavior as before, nevertheless a 20 km diameter unrealistically hot (*ΔT* = 200 °C) thermal anomaly composed of hydrated mantle material can propagate through the entire crust (Fig. 3). Smaller and colder anomalies can ascend through the lower crust, but they eventually become stagnant at mid-crustal levels. The last set of simulations contains the second and the third thermal pulse inserted after 1000 and 1500 numerical time steps, respectively. In this case models with thermal anomalies of 10 km in diameter are not able to penetrate the crust, independent of the temperature anomaly and the rate of crustal extension (Fig. S8). Increasing the anomaly size to 15 km we observe a coherent pattern in which partially molten hydrated magmas are able to penetrate the entire crust and reach the surface in less than 1 Myr (Fig. 4; Movies SM1 and SM2). This behavior is noted for high strain rates of 7.927 × 10^–16^ s^−1^ for the entire anomaly temperature domain (100–200 °C). For smaller extensional rates of 6.342 × 10^–16^ s^−1^ or less (Fig. S9) hydrated magmas cannot cross the mid-crustal level in less than 1 Myr of model evolution. Decreasing more the extensional rates to 4.756 × 10^–16^ s^−1^ or less, thermal anomalies remain trapped in the lower crust (Fig. S9) even at high values of *ΔT* (i.e., 200 °C). On the other hand, thermal anomalies of 20 km in size and *ΔT* of 100–180 °C can penetrate the entire crust when the regional extensional rates are in the range of 6.342–7.927 × 10^–16^ s^−1^ (Movie SM3). For smaller rates (i.e., < 6.342 × 10^–16^ s^−1^) the thermal energy of the three anomalies is dissipated through lateral propagation at the base of lithosphere (Fig. 5). Other model combinations (i.e., high extensional rates and *ΔT)* predict a catastrophic surface event where unrealistically large quantities of partially molten hydrated mantle are brought to the surface (Fig. S10).

## Crustal and surface deformation

**Figure 6:**
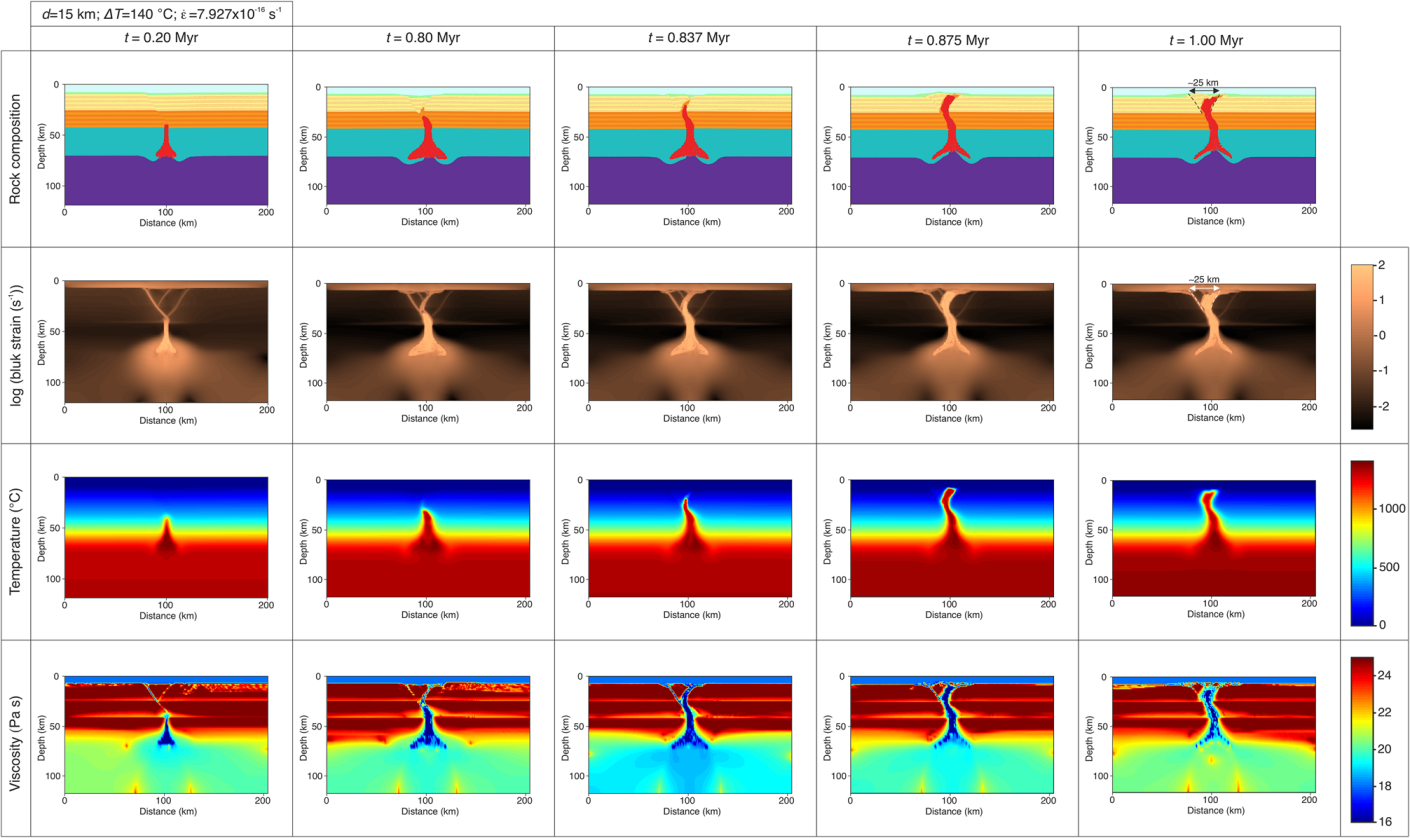
Time series showing modeling results (rock composition, strain rate, temperature and viscosity) for a simulation with three thermal anomalies of 15 km in diameter, a *ΔT* = 140 °C and extensional background strain rate of 7.927 × 10^–16^ s^−1^.

Depending on temperature, pressure and stress, crustal rocks show different deformation organization, and rocks deform irreversibly by both viscous and plastic deformation. For example, at low temperature and pressure rocks tend to behave localized brittle, but increasing pressure (i.e., depth) and temperature plastic deformation is the main deformation mechanism. The upper part of the modeling domain is dominated by plastic deformation. This is because our models consider a visco-elasto-plastic rheology where an absolute shear stress limit σ_yield_ exists for the continental crust (see Supplementary Information). After reaching this limit plastic yielding occurs, and although like viscous deformation, plastic yielding is irreversible, the pattern of deformation is particularly different with the formation of a series of shear bands. This style of deformation produces fracture zones and shear zones in natural rock complexes^38^. We show that when the heterogeneous modeling domain is not subjected to uniaxial extension, no hot mantle intrusion (dry or hydrated) can reach the surface (Fig. S2–S7). However, upon extension, significant strain starts to localize around base of the continental crust where we allow the magmatic channel to penetrate a couple of kilometers (Fig. S1A). The top of the magmatic channel acts as a weak inclusion that concentrate the formation of shear bands which propagate to the surface of the model (Fig. 6). This is consistent with Jacquey and Cacace^39^ that shows the origin and formation of shear bands strongly depends on the presence of material heterogeneities. In our models the localization of deformation is initially concentrated in a couple of symmetrical shear bands, but as the model evolves more narrow share bands develop from the initial ones (Fig. 6) and the deformation patten becomes asymmetrical. The bands formed around the base of the lower crust are oriented approximately 45°, but become more steeper in the upper crust due to different mechanical parameters (i.e., internal cohesion and angle of friction, see Table S1). These secondary shear bands are controlled by the limit between the upper and lower continental crust, and in time only several shear bands remain active and eventually allow hot mantle to penetrate towards the surface.

In terms of surface deformation, this is mainly controlled by the localization of deformation within the continental crust. One of the best models that reassemble LHVF is presented in Fig. 6, and include the succession of three thermal anomalies of 15 km in diameter with *ΔT* of 140 °C. Similar results are obtained when *ΔT* is reduced to 120 °C or less (Movies SM1 and SM2). The hot low viscosity mantle material arrives at the surface in some 0.875 Myr and is followed by a subsequent cooling and collapse of the central uplifted area. The presence of weak and hot mantle material at the base of the crust helps maintaining the localized deformation within the central part of the modeling domain. During extension and magma intrusion propagation two stages are clearly seen: (1) normal faulting of the upper and lower continental crust (Fig. 6) and (2) growth and localization of deformation associated with the propagation of a hydrated mantle towards the surface (Fig. 6). Extensional deformation associated with normal faulting in the early stages of our experiments initially starts at the bottom of the continental crust and propagates in a series of shear bands towards the surface where it covers a 50 km wide area. It is followed by a period where plastic deformation is focused on only a couple of shear bands and then the partially molten mantle material migrates upward the surface in a relatively short period of time. The first part of these experiments is characterized by topography development in a form specific for extensional models where we observe the formation of a topographic low (Fig. 5). However, the topographic low is reversed for models where mantle material succeeds in penetrating the entire crust. The width of the uplifted region is confined between two main share bands located some 25 km apart.

## Discussion and conclusions

Although several caldera-forming collapses are recorded in the last century^8^, the origin of large volumes of magma supplied from below is still not fully understood. Although some of these calderas are located in the vicinity of active subduction systems, the connection between subduction and calderas remained somehow elusive. Within this complex framework, investigating the geodynamic origin of LHVF represents a good opportunity to advance more towards a better assessment of caldera source. Our strategy involves high-resolution two-dimensional thermomechanical numerical simulations (see Methods) of magma intrusions to better understand the formation of LHVF caldera. Given the presence of multiple eruption events at LH caldera, including pre-calderic, co-calderic and post-calderic events (Fig. 1), our main conjecture is based on the existence of one or more thermal mantle anomalies that are able to efficiently propagate through the lithosphere and crust and produce the LHVF caldera. Since the TMVB is characterized by an extension rate of ~ 3% and our models are 200 km wide, we perform numerical simulations with extensional background strain rates ranging from of 0 (no extension) to 7.927 × 10^–16^ s^−1^ and all models are integrated in time maximum 1 Myr. The first set of models involve the presence of one or more thermal anomalies composed of dry and hydrated mantle material. The thermal anomalies are appended in the model from the beginning and at specific time steps, and for numerical modeling convenience placed at the base of lithosphere. Modeling results show that dry hot mantle material is not able to propagate through the entire crust, and the partially molten material remains trapped in the lower crust. Actually, irrespective of the background strain rates models with dry or hydrated mantle thermal anomalies show a similar behavior. This points to the critical importance of regional tectonic stresses^18,40^ in generating the necessary conditions for surface manifestation of thermal anomalies. The first models that predict magma propagation to the surface include a minimum of time-separated (0.37 Myr) two large (20 km in diameter) hot (∆T = 200 °C) thermal pulses and an extensional background stress of 7.927 × 10^–16^ s^−1^ which represent our upper limit (Fig. 3). However, in terms of temperature anomaly this is unrealistically high^41,42^. Adding another thermal pulse (of the same size and ΔT as the initial one) predict results where hydrated mantle material can arrive at the surface in less than 1 Myr (Fig. 4 and 5). However, only anomalies of 15 or more in size show successful results and models of this type with 10 km anomalies are ineffective (Fig. S8). Also, the minimum extensional background stress required for suitable results are in the range of 6.342–7.927 × 10^–16^ s^−1^ (Fig. 4). The temperature anomalies for these simulations are in the range of 100–140 °C. Moreover, we observed that it is essential that thermal pulses arrive at the base of lithosphere within a time frame that facilitates a rapid ascent and avoid the extensive cooling before reaching the surface of the crust. In terms of minimum estimates, our models predict that hydrated magma is able to rise to the surface when the extensional background stress is no less than 6.342 × 10^–16^ s^−1^, anomaly diameter minimum of 15 km and ∆T ≥ 100 °C (Movies SM1 and SM2). The need for a minimum of three time-separated thermal pulses to form a volcanic structure of LHVF dimensions (Fig. 6) reflects the multidimensional source nature of the asthenospheric origin of LHVF and possible to other volcanic calderas located in a similar tectonic setup.

A possible explanation for our modeling results should consider the presence of the Cocos slab below LHVF, although currently located at depths in excess of 300 km. Our model was proposed taking account of the geodynamic evolution in the area that includes the detachment in the Cocos slab^10,20^ and its subsequent rollback^8,43^, which generated extension since the late Miocene along the TMVB^9,40,44^. Lateral propagation of slab detachment along the TMVB is based on evidence of an eastward-migrating mafic volcanism^10^, that arrived at ~ 6 Ma in the region of LHVF^10^. At the same time, the geochemical characteristics of magmas associated with LHVF units show strong variations in their generation processes in a period of approximately 1 Myr^26^. This change is probable associated with the composition of the mantle wedge^26^. Low values of Th/Nb and La/Sm (normalized to chondrite) in the pre-calderic phase and the volcanic basement reflect that there was no contribution of material from the slab^26^, but they are compositionally associated with intraplate magmatism, as also shown by isotope signature, like the ratio of ${{\text{Sr}}}^{87}/{{\text{Sr}}}^{86}$ and ${{\text{Nd}}}^{143}/{{\text{Nd}}}^{144}$^15–17^. However, a high Ba/Th value determines that there are fluids from the slab that contribute to the formation of partial melts^26^.

**Figure 7:**
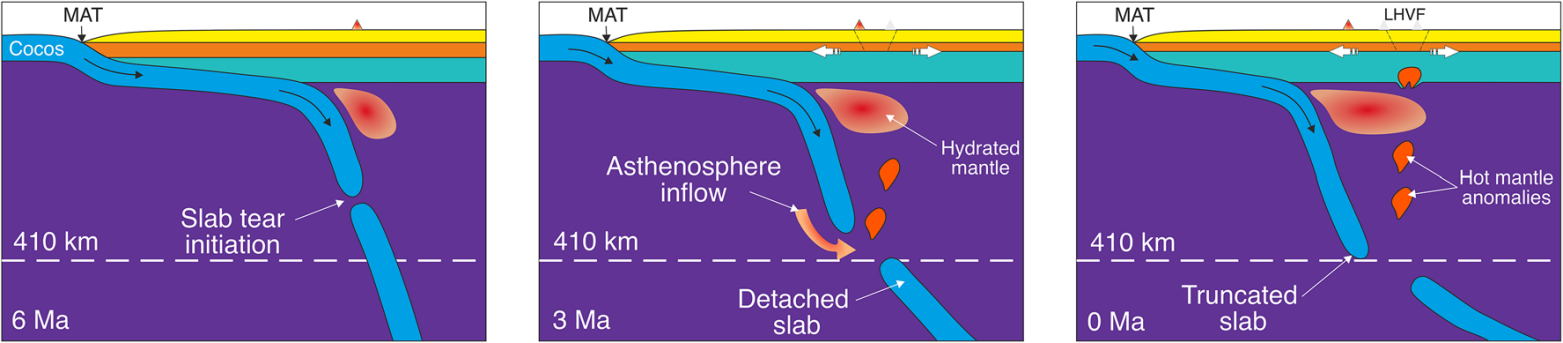
Proposed evolution of subduction of the Cocos plate beneath Mexico in the region of LHVF. Slab tear initiation^10^. The subsequent widening of slab tear coupled with slab rollback allows hot (*ΔT* = 100–140 °C) asthenospheric material flow through the slab gap and migrate upwards where might interact with the hydrated mantle wedge. A chain type of several hydrated mantle plumes (of 15–20 km in size) arrives at the base of lithosphere where start to pierce the continental lithosphere and crust and eventually create LHVF. White divergent arrows at the base of the crust illustrate the continental extension. *MAT* - Middle America Trench.

On the contrary, the high values of La/Sm (normalized to chondrite) and Th/Nb indicate a contribution of slab-molten sediments^26,45–47^, which is observed in the calderic and post-calderic phases^26^. Partial melting of the sediments is possible due to the retreat of the slab, which can create the necessary conditions of temperature and pressure for its formation^48^. These geochemical characteristics show a greater contribution of subduction material in the calderic and post-calderic phases regarding the pre-calderic phase and the volcanic basement. In the quest for offering a plausible geodynamic origin of LHVF, we remark that recent seismological studies^25^ show the accumulation of partial melting under the TMVB in the mantle-crust boundary from the front to the back of the volcanic arc. This observation can explain how magmas associated with the subducted slab are located as far as in the rear part of the arc. On the other hand, there are studies that indicate that the magmas that formed LHVF are not actually associated with the Cocos slab and are generated mainly by decompression associated with the regional extension triggered by the rollback^32^. In this context the geochemical signature of the calderic and post-calderic units with contribution of sediments from the slab are explained by crustal assimilation and not necessarily related to the slab^32,49^. However, we argue that the extension rate alone in the eastern zone of the TMVB is insufficient to generate partial decompression melt^36^. Based on these lines of evidence we interpret the origin of LHVF in terms of the relationship between subduction, mantle flow and thermal anomalies. It is worth mentioning that slabs act as a cold shield that, when fragmented, allows the ascent of hot material into the asthenospheric wedge^10,48^. Other numerical models indicate that slab detachment causes a significant local temperature increase due to the rise of the hot asthenosphere^50^. Based on these lines of evidence we propose that the geodynamic origin of LHVF is related with thermal instabilities generated by the detachment and subsequent rollback of the Cocos slab initiated at ~ 6 Ma^10,20^ (Fig. 7). They flow from deep (> 300 km depth) asthenospheric sub-slab regions through the Cocos slab gap into the mantle wedge above. Given the adiabatic gradient of 0.5 °C/km, at depths below 300 km the mantle temperature is over 100–120 °C more than at the base of the lithosphere (considered at 70 km depth in our simulations). This is consistent with our predictions where temperature anomalies should be over 100 °C in order to propagate through the continental lithosphere and crust. Sub slab asthenospheric thermal anomalies migrate upwards and interact with the hydrated mantle wedge creating hydrated melt before reaching the base of the lithosphere. This can explain why our simulations predict that only hydrated mantle material can penetrate through the low viscosity shear bands created by regional background extension. We conclude that the best scenario for LHVF origin is the combination of several favorable key parameters, as a chain of several mantle hydrated thermal anomalies (d > 15 km and (*ΔT* > 100 °C) coupled with a regional background extension rate of < 3%. Probably the most important outcome of this study is the finding that volcanic calderas in general, and LHVF in particular, are actually formed not by a single mantle source, but rather by multiple mantle anomalies that thermally batter the continental crust until fails along a preexistent fault system and eventually erupt vigorously at the surface.

## Methods: numerical modeling strategy, setup and boundary conditions

We employed the numerical approach described by Gerya (2010) to solve the 2D equations of continuity, momentum and heat conservation with the finite difference method coupled with the cell marker technique and the multigrid method^51^ (see Supplementary Information). The mechanical boundary conditions are as follows: the top and bottom boundaries are free slip; and the lateral boundaries are free slip or have a prescribed outward velocity (horizontal extension). In addition, we used erosion and transportation processes, a visco-elasto-plastic rheology, and compute melt fraction and latent heat at given pressure for each rock layer (Fig. S1). We use a Maxwell visco-elastic rheology which is based on the assumption that both viscous and elastic deformations are happening under the same applied deviatoric stress. The initial model size is 120 × 200 km (the numerical configuration is shown in Fig. S1A), and the initial configuration setup has a continental lithosphere and a part of the asthenosphere (see Table S1 for detailed materials properties). We used a 35 km thickness (uniformly divided into lower crust and upper crust) for the continental crust (including a 2 km thick sedimentary layer), similar to the study of Ferrari et al.^8^ for Los Humeros area. In order to correctly represent the internal free surface, the most superficial layer is a 7 km thick “sticky air” layer^51,52^. A 2D staggered and irregularly spaced (with 1 km grid resolution in the center of the model) numerical grid is used. We use a high resolution of 1 km in both horizontal and vertical directions in the top middle of the modeling domain where strongly localized deformation along faults is expected (Gerya, 2019). To facilitate the upward propagation of thermal anomalies, we include a 3 km wide lithospheric channel with the same composition and thermal proprieties as the thermal anomalies. We argue that these channels can be formed naturally in an early magmatic stage beneath LHVF due to the rapid rise of hot mobile fluids, which are differentiated products on the anomaly^51^. Thermal anomalies are composed of dry or hydrated mantle and have different spherical size and temperature excess. They are located initially below the lithosphere-asthenosphere boundary and our modeling strategy also includes a dynamic insertion (at different simulation stages) of thermal anomalies during the numerical simulations. We used a total of approximatively 600,000 random Lagrangian markers according to a computed velocity field and using a Runge–Kutta fourth-order advection scheme to track lithological evolution through time^20,51^. The initial temperature is uniformly stratified, 0 °C at the surface and 1327 °C at the bottom. The adiabatic temperature gradient for the asthenospheric mantle is 0.5 °C/km^3^.

### Supplementary Information

Supplementary Video 1.

Supplementary Video 2.

Supplementary Video 3.

Supplementary Information 1.

## Data Availability

The datasets used and/or analysed during the current study are available from the corresponding author on reasonable request.

## References

[1] Bercovici, D. Mantle dynamics. In *Treatise on Geophysics* (Vol. 7) (ed. Schubert, G.) (2007).

[2] Schubert G, Turcotte D, Olson P (2004). Mantle Convection in the Earth and Planets.

[3] Turcotte D, Schubert G (2014). Geodynamics (Third).

[4] Apperson D (1991). Stress fields of the overriding plate at convergent margins and beneath active volcanic arcs. Science.

[5] Straub SM, Zellmer GF (2012). Volcanic arcs as archives of plate tectonic change. Gondwana Res.

[6] Aguirre-Díaz G, McDowell F (1999). Volcanic evolution of the Amealco caldera, central Mexico. Geol Soc Am.

[7] Carrasco-Núñez G, Mccurry M, Branney M, Norry M, Willcox C (2018). Complex magma mixing, mingling, and withdrawal associated with an intra-Plinian ignimbrite eruption at a large silicic caldera volcano : Los Humeros of central Mexico. Bull Geol Soc Am.

[8] Ferrari L, Orozco-Esquivel T, Manea V, Manea M (2012). The dynamic history of the trans-Mexican volcanic belt and the Mexico subduction zone. Tectonophysics.

[9] Alaniz S, Nieto-Samaniego Á, Ferrari L (1999). Effect of strain rate in the distribution of monogenetic and polygenetic volcanism in the transmexican volcanic belt. Geology.

[10] Ferrari L (2004). Slab detachment control on mafic volcanic pulse and mantle heterogeneity in central Mexico. Geol. Soc. Am..

[11] Portner DE, Hayes GP (2018). Incorporating teleseismic tomography data into models of upper mantle slab geometry. Geophys. J. Int..

[12] Amante, C., & Eakins, B. W. ETOPO1 1 arc-minute global relief model: procedures, data sources and analysis, in *NOAA Technical Memorandum NESDIS NGDC-24*, Issue March, p. 19. 10.1594/PANGAEA.769615 (2009).

[13] Cavazos-Álvarez JA, Carrasco-Núñez G (2020). Anatomy of the Xáltipan ignimbrite at Los Humeros volcanic complex; the largest eruption of the trans-Mexican volcanic belt. J. Volcanol. Geotherm. Res..

[14] Carrasco-Núñez G (2022). Assembly and development of large active calderas hosting geothermal systems: Insights from Los Humeros volcanic complex (Mexico). J. South Am. Earth Sci..

[15] Verma S (1984). Alkali and alkaline earth element geochemistry of Los Humeros Caldera, Puebla, Mexico. J. Volcanol. Geotherm. Res..

[16] Verma S (2000). Geochemical evidence for a lithospheric source for magmas from Los Humeros caldera, Puebla, Mexico. Chem. Geol..

[17] Verma S (1983). Magma genesis and chamber processes at Los Humeros caldera, Mexico-Nd and Sr isotope data. Nature.

[18] García-Palomo A (2018). NW-SE pliocene-quaternary extension in the Apan-Acoculco region, eastern trans-Mexican volcanic belt. J Volcanol Geotherm Res.

[19] Rodríguez SR, Morales-Barrera W, Layer P, González-Mercado E (2010). A quaternary monogenetic volcanic field in the Xalapa region, eastern trans-Mexican volcanic belt: Geology, distribution and morphology of the volcanic vents. J Volcanol Geotherm Res.

[20] Ferrari L (2005). Geology, geochronology and tectonic setting of late Cenozoic volcanism along the southwestern Gulf of Mexico: The eastern alkaline province revisited. J. Volcanol. Geotherm. Res..

[21] Corbo-Camargo F (2020). Shallow structure of los humeros (LH) caldera and geothermal reservoir from magnetotellurics and potential field data. Geophys J Int.

[22] Gutiérrez-Negrín, L. C. A., & Izquierdo-Montalvo, G. Review and update of the main features of the Los Humeros geothermal field, Mexico, in *World Geothermal Congress*, 25–29 (2010).

[23] Carrasco-Núñez G (2018). Reappraisal of Los Humeros volcanic complex by new U/Th zircon and 40Ar/39Ar dating: Implications for greater geothermal potential. Geochem. Geophys. Geosyst..

[24] Carrasco-Nuñez G (2017). Geologic map of Los Humeros volcanic complex and geothermal field eastern trans-Mexican volcanic belt. Terra Digit..

[25] Castellanos JC, Clayton RW, Pérez-Campos X (2018). Imaging the eastern trans-Mexican volcanic belt with ambient seismic noise: Evidence for a slab tear. J. Geophys. Res. Solid Earth.

[26] Créon L, Levresse G, Carrasco-Nuñez G, Remusat L (2018). Evidence of a shallow magma reservoir below Los Humeros volcanic complex: Insights from the geochemistry of silicate melt inclusions. J. South Am. Earth Sci..

[27] Bonini M (2021). modeling intra-caldera resurgence settings: Laboratory experiments with application to the Los Humeros volcanic complex (Mexico). J. Geophys. Res. Solid Earth.

[28] Deb P, Giordano G, Shi X, Lucci F, Clauser C (2021). An approach to reconstruct the thermal history in active magmatic systems: Implications for the Los Humeros volcanic complex, Mexico. Geothermics.

[29] Urbani S (2020). Estimating the depth and evolution of intrusions at resurgent calderas: Los Humeros (Mexico). Solid Earth.

[30] Granados-Chavarría I, Calò M, Figueroa-Soto Á, Jousset P (2022). Seismic imaging of the magmatic plumbing system and geothermal reservoir of the Los Humeros caldera (Mexico) using anisotropic shear wave models. J. Volcanol. Geotherm. Res..

[31] Lucci F (2020). Anatomy of the magmatic plumbing system of Los Humeros caldera (Mexico): Implications for geothermal systems. Solid Earth.

[32] Gómez-Tuena A, LaGatta AB, Goldstein SL, Ortega-Gutie F, Carrasco-Núñez G (2003). Temporal control of subduction magmatism in the eastern trans-Mexican volcanic belt: Mantle sources, slab contributions, and crustal contamination. Geochem. Geophys. Geosyst..

[33] Fitz-Díaz E, Lawton TF, Juárez-Arriaga E, Chávez-Cabello G (2018). The cretaceous-paleogene Mexican orogen: Structure, basin development, magmatism and tectonics. Earth-Sci. Rev..

[34] Campos-Enriquez J, Garduño-Monroy V (1987). The shallow structure of Los Humeros and Las Derrumbadas geothermal fields, Mexico. Geothermics.

[35] Norini G (2019). The structural architecture of the Los Humeros volcanic complex and geothermal field. J. Volcanol. Geotherm. Res..

[36] Ferrari L, Petrone C, Francalanci L (2001). Generation of oceanic-island basalt—type volcanism in the western trans-Mexican volcanic belt by slab rollback, asthenosphere infiltration, and variable flux melting. Geology.

[37] Manea VC, Manea M, Kostoglodov V, Sewell G (2005). Thermo-mechanical model of the mantle wedge in central Mexican subduction zone and a blob tracing approach for the magma transport. Phys. Earth Planet. Inter..

[38] Gerya T (2019). Introduction to numerical geodynamic modelling.

[39] Jacquey AB, Cacace M (2020). Multiphysics modeling of a brittle-ductile lithosphere: 2. Semi-brittle, semi-ductile deformation and damage rheology. J. Geophys. Res. Solid Earth.

[40] Suter M, Martínez ML, Legorreta OQ, Martínez MC (2001). Quaternary intra-arc extension in the central trans-Mexican volcanic belt. Bull Geol Soc Am.

[41] Schutt DL, Dueker K (2008). Temperature of the plume layer beneath the Yellowstone hotspot. Geology.

[42] White RS, Bown JW, Smallwood JR (1995). The temperature of the Iceland plume and origin of outward- propagating V-shaped ridges. J. Geol. Soc..

[43] Manea V, Manea M, Ferrari L (2013). A geodynamical perspective on the subduction of Cocos and Rivera plates beneath Mexico and central America. Tectonophysics.

[44] Suter M, Lopez-Martinez M, Aguirre-Diaz G, Farrat E (1995). The Acambay graben: Active intraarc extension in the trans-Mexican volcanic belt, Mexico. Tectonics.

[45] Ayers JC, Dittmer SK, Layne GD (1997). Partitioning of elements between peridotite and H_2_O at 2.0–3.0 GPa and 900–1100°C, and application to models of subduction zone processes. Earth Planet. Sci. Lett..

[46] Brenan JM, Shaw HF, Ryerson FJ, Phinney DL (1995). Mineral-aqueous fluid partitioning of trace elements at 900°C and 2.0 GPa: Constraints on the trace element chemistry of mantle and deep crustal fluids. Geochim. Cosmochim. Acta.

[47] Johnson MC, Plank T (1999). Dehydration and melting experiments constrain the fate of subducted sediments. Geochem. Geophys. Geosyst..

[48] Orozco-Esquivel T, Petrone CM, Ferrari L, Tagami T, Manetti P (2007). Geochemical and isotopic variability in lavas from the eastern trans-Mexican volcanic belt: Slab detachment in a subduction zone with varying dip. Sci. Direct.

[49] Márquez A, Verma SP, Anguita F, Oyarzun R, Brandle JL (1999). Tectonics and volcanism of Sierra Chichinautzin: Extension at the front of the central trans-Mexican volcanic belt. J. Volcanol. Geotherm. Res..

[50] Van de Zedde DMA, Wortel MJR (2001). Shallow slab detachment as a transient source of heat at midlithospheric depths. Tectonics.

[51] Gerya T (2010). Introduction to Numerical Geodynamic Modelling.

[52] Manea M, Manea V, Ferrari L, Orozco-Esquivel T (2019). Delamination of sub-crustal lithosphere beneath the Isthmus of Tehuantepec, Mexico: Insights from numeric modelling. J. Geodyn..

