# Supplementary material for: The geodynamic origin of Los Humeros volcanic field in Mexico: insights from numerical simulations
Source: Sci Rep. 2023 Dec 14;13:22284. doi: 10.1038/s41598-023-49292-x (PMC10721639; doi:10.1038/s41598-023-49292-x)
Supplement: Supplementary file 4 — Supplementary Information 1. [file 41598_2023_49292_MOESM4_ESM.docx]

Supplementary Information for

The geodynamic origin of Los Humeros Volcanic Field in Mexico: insights from numerical simulations

A. Bayona^1,2^, V.C. Manea^1,2*^, M. Manea^1,2^, S. Yoshioka^2,3^, E. Moreno^2^ and N. Suenaga^2^

^1^-Computational Geodynamics Laboratory, Centro de Geociencias, Universidad Nacional Autónoma de México, Campus Juriquilla, Querétaro, 76230, México.

^2^- Research Center for Urban Safety and Security, Kobe University, Kobe 657-8501, Japan.

^3^-Department of Planetology, Graduate School of Science, Kobe University, Kobe 657-8501, Japan.

*-Corresponding author: [vlad@geociencias.unam.mx](mailto:vlad@geociencias.unam.mx)

**Contents of this file**

1. Supplementary model settings and parameters
2. Supplementary model simulations
3. Supplementary animations
4. Supplementary References

**Introduction**

The Supplementary Information provides the supplementary model settings (Fig. S1), supplementary model simulations (Fig. S2-S10), supplementary movies (SM1-SM3), supplementary tables (1 and 2), and supplementary references.

1. **Supplementary model settings and parameters**

We implemented time-dependent 2D thermomechanical models, which use different initial conditions of mantle thermal anomalies (*d* and *ΔT*) in order to allow mantle material to penetrate the lithosphere and crust and generate structures (i.e., caldera rim faulting system) of similar dimensions as Los Humeros Volcanic Field (LHVF) located on the eastern edge of the Trans-Mexican Volcanic Belt (TMVB).

- 1. **Governing equations**

The governing equations are the conservation of mass (Eq. 1), momentum (Eq. 2), and energy (Eq. 3) (Gerya, 2010).

$\frac{D\rho}{Dt}+\rho\nabla\cdot v=0$ (Eq. 1)

$\frac{\partial\sigma_{ij}}{\partial x_{j}}+\rho g_{i}= \rho\frac{Dv_{i}}{Dt}$ (Eq. 2)

$\rho C_{p}\frac{DT}{Dt}= -\frac{\delta q_{i}}{\delta x_{i}}+H$ (Eq. 3)

where $\rho$ is the density, $v$ is the velocity, *t* is the time, *i* and *j* are the coordinates indices, *σ_ij_* is the stress,$g$ is the gravity acceleration, *x_i_* and *x_j_* are the spatial coordinates, *q* is the heat flux, the *i* -index is the sum of heat flux components ${(q}_{x} \& q_{y})$, $C_{p}$ is the heat capacity and *H* is the volumetric heat production.

- 1. **Visco-elasto-plastic rheology**

We used a visco-elasto-plastic rheology in the numerical models. This is formulated by decomposing the deviatoric strain rate ($\dot{Ɛ}_{ij}^{'}$) into its three components (Gerya & Yuen, 2007):

$$\dot{Ɛ}_{ij}^{'}= \dot{Ɛ}_{ij(viscous)}^{'}+ \dot{Ɛ}_{ij(plastic)}^{'}+\dot{Ɛ}_{ij(elastic)}^{'}$$

where

$$\dot{Ɛ}_{ij(viscous)}^{'}=\frac{1}{2ղ}\sigma_{ij}^{'},$$

$$\dot{Ɛ}_{ij(elastic)}^{'}=\frac{1}{2\mu}\frac{D\sigma_{ij}^{'}}{Dt},$$

$\dot{Ɛ}_{ij(plastic)}^{'}=0 for \sigma_{II}<\sigma_{yield}, \dot{Ɛ}_{ij(plastic)}^{'}= \frac{\partial G_{plastic}}{\partial\sigma_{ij}^{'}}= X\frac{\sigma_{ij}^{'}}{2\sigma_{II}}\mathrm{for}\sigma_{II}=\sigma_{yield},$

$$G_{plastic}= \sigma_{II},$$

$$\sigma_{II}= \sqrt{\frac{1}{2}\sigma_{ij}^{'2}},$$

$$\sigma_{yield}=C+\sin\left( \varphi\right)P,$$

$\sin\left( \varphi\right)=\sin\left( \varphi_{dry} \right)\lambda,$

*λ* = $\frac{P_{fluid}}{P_{solid}},$

where $ղ$ it the viscosity,$\sigma_{ij}^{'}$ is the component of the deviatoric stresses, $\sigma_{yield}$is the elastic-plastic limit, $\mu$ is the shear modulus, $\sigma_{II}$ is the second invariant of the deviatoric stresses, $G_{plastic}$ is the plastic flow potential, $C$ is the cohesion (residual strength at P = 0), $\varphi$ is effective internal friction angle ($\varphi_{dry}$ stands for dry rocks), λ is the pore fluid pressure factor, $P_{solid}$ is the mean stress and $P_{fluid}$ is the pore fluid pressure.

- 1. **Phase equations**

We apply phase equations that relate pressure and temperature according to the different materials used in the simulations (upper crust, lower crust, and mantle) to calculate the partial melt. Partial melt is calculated from the following equation (Gerya, 2010):

$$Partial melt= \frac{(T-t_{s})}{(t_{l}-t_{s})}$$

where *T* is the current temperature, *t_s_* is the solid phase temperature and *t_l_* is the liquid temperature of the material. If the partial melt number is < 0, it is taken 0 as the value. If the partial melt number is > 1, it is chosen 1 as the value. Partial melt ranges from 0 to 1, in which 0 means 100% solid and 1 means 100% liquid.

- - 1. **Upper crust**

The following equation was used to obtain the solid phase (*t_s_*) curve:

Pressure (*P*) < 1200 MPa:

$$ts= 889+\frac{17900}{\left( P+54 \right)}+ \frac{20200}{\left( P+54 \right)^{2}}$$

Pressure (*P*) $\geq$ 1200 MPa:

$$t_{s}= 831+(0.06P)$$

The liquid phase curve (*t_l_*) was calculated with the following equation:

$$t_{l}= 1262+0.09P$$

where *t_s_, t_l_* and *P* are the variables, and the other numbers are constant.

- - 1. **Lower crust**

The following equation was used to obtain the solid phase (*t_s_*) curve:

Pressure (*P*) < 1600 MPa:

$$t_{s}=973-\frac{70400}{(P+354)}+ \frac{77800000}{{(P+354)}^{2}}$$

Pressure (*P*) $\geq$ 1600 MPa:

$$t_{s}= 935+{0.0035P+(0.0000062P)}^{2}$$

The liquid phase curve (*t_l_*) was calculated with the following equation:

$$t_{l}=1423+0.105P$$

where *t_s_, t_l_* and *P* are the variables, and the other numbers are constant.

- - 1. **Mantle**

The following equation was used to obtain the solid phase (*t_s_*) curve:

Pressure (*P*) $<$ 10000 MPa:

$$t_{s}=1394+0.132899P-(0.000005104){(P)}^{2}$$

Pressure (*P*) $\geq$ 10000 MPa:

$$t_{s}=2212+(0.030819\left( P-10000 \right))$$

The liquid phase curve (*t_l_*) was calculated with the following equation:

$$t_{l}=2073+(0.114P)$$

where *t_s_, t_l_* and *P* are the variables, and the other numbers are constant.

- 1. **Surface erosion and deposition**

The next equations were used to calculate the surface erosion and deposition.

$$Surface erosion=(Erosion rate)/(1000*365.25*24*3600)$$

$$Surface deposition=(Surface erosion*\left( 10*1000 \right)^{2})/(10*1000)$$

We used an *Erosion rate* of 1 mm/year in all numerical models.

- 1. **Rock composition**

The rock composition of the model corresponds to: (1) granodiorites with a layer of superficial sedimentary rocks in the upper crust, and (2) diorites in the lower crust. Furthermore, the composition of the thermal anomaly is either dry or hydrated asthenospheric mantle. The dimensions and other parameters used in the models are specified in Supplementary Tables 1 and 2.

| 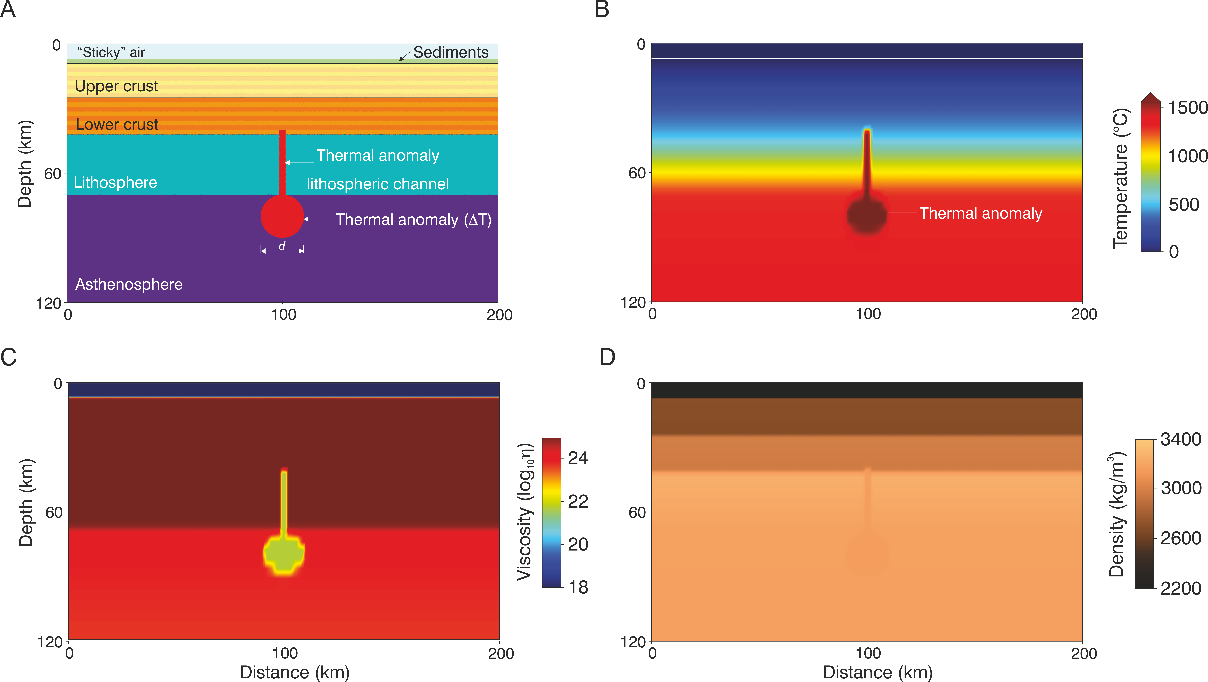 |
| --- |
| **Figure S1.** Initial numerical model setup: A) Rock composition, B) Temperature, C) Viscosity, D) Density. Anomaly diameter *d* is varied from 10 km to 20 km (25 km), and temperature anomaly *ΔT* from 100 °C to 200 °C (250°C). The lithospheric channel has the same rock composition and *ΔT* as the thermal anomaly and an initial diameter of 3 km in all simulations. Upper and lower crusts are marked in rock composition with colored stripes only to better observe the deformation pattern. |

**Supplementary Table 1.** Material properties used in numerical experiments (Bittner & Schmeling, 1995; Clauser & Huenges, 1995; Gerya, 2010; Hess, 1989; Hirschmann, 2000; Hofmeister, 1999; Johannes, 1985; Ranalli, 1995; Schmidt & Poli, 1998, 2002; Turcotte & Schubert, 2002).

| **LAYERS (depth from model top)** | **PARAMETERS** | | | | | | | | | |
| --- | --- | --- | --- | --- | --- | --- | --- | --- | --- | --- |
|  | **Density (kg/**$\mathbf{m}^{\mathbf{3}}$**)** | **Thermal expansion (1/K)** | **Compre-ssibility (1/Pa)** | **Melt density (kg/**$\mathbf{m}^{\mathbf{3}}$**)** | **Shear module (Pa)** | **Heat capacity (J/Kg)** | **Thermal conductivity (W/m/K)** | **Effective internal friction angle**  **(sin(** $\boldsymbol{\varphi}$**))** | **Radiogenic heat production**  **(W/**$\mathbf{m}^{\mathbf{3}}$**)** | **Cohesion**  **(Pa)** |
| **Sticky air (<7km)** | 1,000 | 0 | 0 | - | 1.00E+20 | 3,000 | 300 | 0 | 0 | 0 |
| **Sedimentary rocks**  **(≥7km-9km)** | 2,700 | 3.00E-05 | 1.00E-11 | 2.40E+03 | 1.00E+10 | 1.00E+03 | 6.40E-01 | 0.2 | 2.00E-06 | 1.00E+06 |
| **Upper continental crust**  **(≥9km-25km)** | 2.70E+03 | 3.00E-05 | 1.00E-11 | 2.40E+03 | 1.00E+10 | 1.00E+03 | 6.40E-01 | 0.2 | 1.00E-06 | 1.00E+06 |
| **Lower continental crust**  **(25km-42km)** | 3.00E+03 | 3.00E-05 | 1.00E-11 | 2.70E+03 | 2.50E+10 | 1.00E+03 | 1.18E+00 | 0.2 | 5.00E-07 | 1.00E+06 |
| **Lithospheric mantle**  **(≥42km-70km)** | 3.30E+03 | 3.00E-05 | 1.00E-11 | 2.70E+03 | 6.70E+10 | 1.00E+03 | 7.30E-01 | 0.6 | 2.20E-08 | 1.00E+06 |
| **Asthenospheric mantle (dry) (≥70km)** | 3.30E+03 | 3.00E-05 | 1.00E-11 | 2.70E+03 | 6.70E+10 | 1.00E+03 | 7.30E-01 | 0.6 | 2.20E-08 | 1.00E+06 |
| **Astenospheric mantle (hydrated) (≥70km)** | 3.30E+03 | 3.00E-05 | 1.00E-11 | 2.40E+03 | 6.70E+10 | 1.00E+03 | 7.30E-01 | 0 | 2.20E-08 | 1.00E+06 |

**Supplementary Table 2.** Constants used in the simulations (Gerya, 2010; Holder et al., 2019; Turcotte D. & Schubert, 2014).

| Gravity (m/s^2^) | 9.81 |
| --- | --- |
| Gas constant (J/mol/K) | 8.314 |
| Minimum viscosity of rocks (Pa s) | 1.00E+16 |
| Maximum viscosity of rocks (Pa s) | 1.00E+25 |
| Potential mantle temperature (K) | 1.553 |
| Viscosity of partially molten rock (Pa s) | 1.00E+16 |

**2. Supplementary model simulations**

In supplementary Figure S2 we show modeling results for a *d*=10-20 km diameter dry mantle thermal anomaly (*ΔT*=100-200 ºC) without background strain rate. All numerical predictions are shown after 1 Myr of evolution. Note that none of the simulations can predict mantle transport through the continental crust and the main deformation is concentrated at the base of lithosphere. Also, small thermal anomalies of 10 km and 15 km in diameter show little of absent deformation for *ΔT*<140 ºC.

| 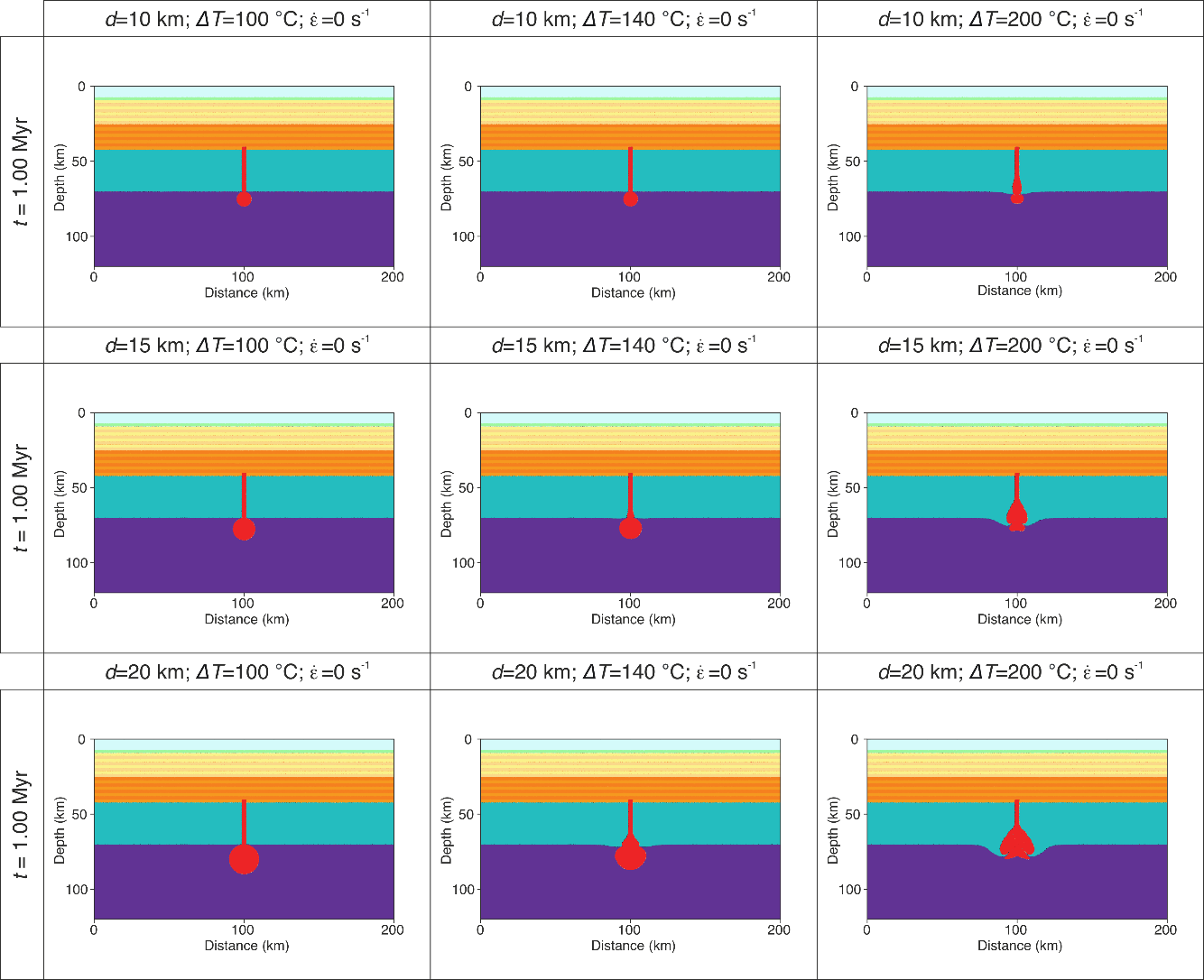 |
| --- |
| **Figure S2.** Evolution of rock composition after 1 Myr for simulations without background strain rate and dry mantle thermal anomalies of 10-20 km in diameter. |

In supplementary Figure S3 we show modeling results for an oversized *d*=25 km dry and hydrated mantle hot thermal anomaly (*ΔT*=250 ºC) without background strain rate. All numerical predictions are shown after 1 Myr of evolution and include only one initial thermal anomaly. Despite the larger size and unusual hot anomaly, no mantle transport through the continental crust is observed. Also, most of the deformation is limited to the first 0.3 Myr, and after this period the thermal anomaly starts equilibrating with the surrounded mantle.

| 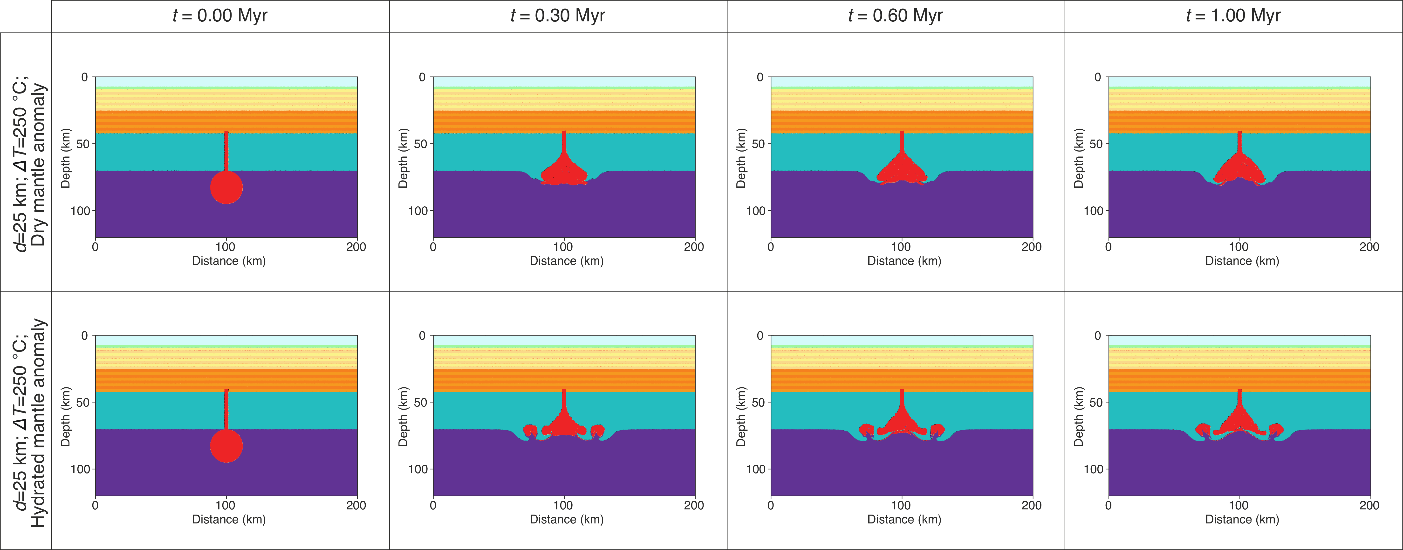 |
| --- |
| **Figure S3.** Evolution of rock composition after 1 Myr for simulations without background strain rate for two models involving a single dry and a hydrated mantle source. The thermal anomaly in both simulation is 25 km in diameter and have *ΔT*=250 ºC. |

In supplementary Figure S4 we show modeling results with an initial thermal anomaly of various sizes *d*=10-20 km composed of hydrated mantle. No background strain rates are applied to the modeling domain and the thermal anomaly includes a temperature excess range of *ΔT*=100-200 ºC. All numerical predictions are shown after 1 Myr of evolution and show no mantle transport through the continental crust.

| 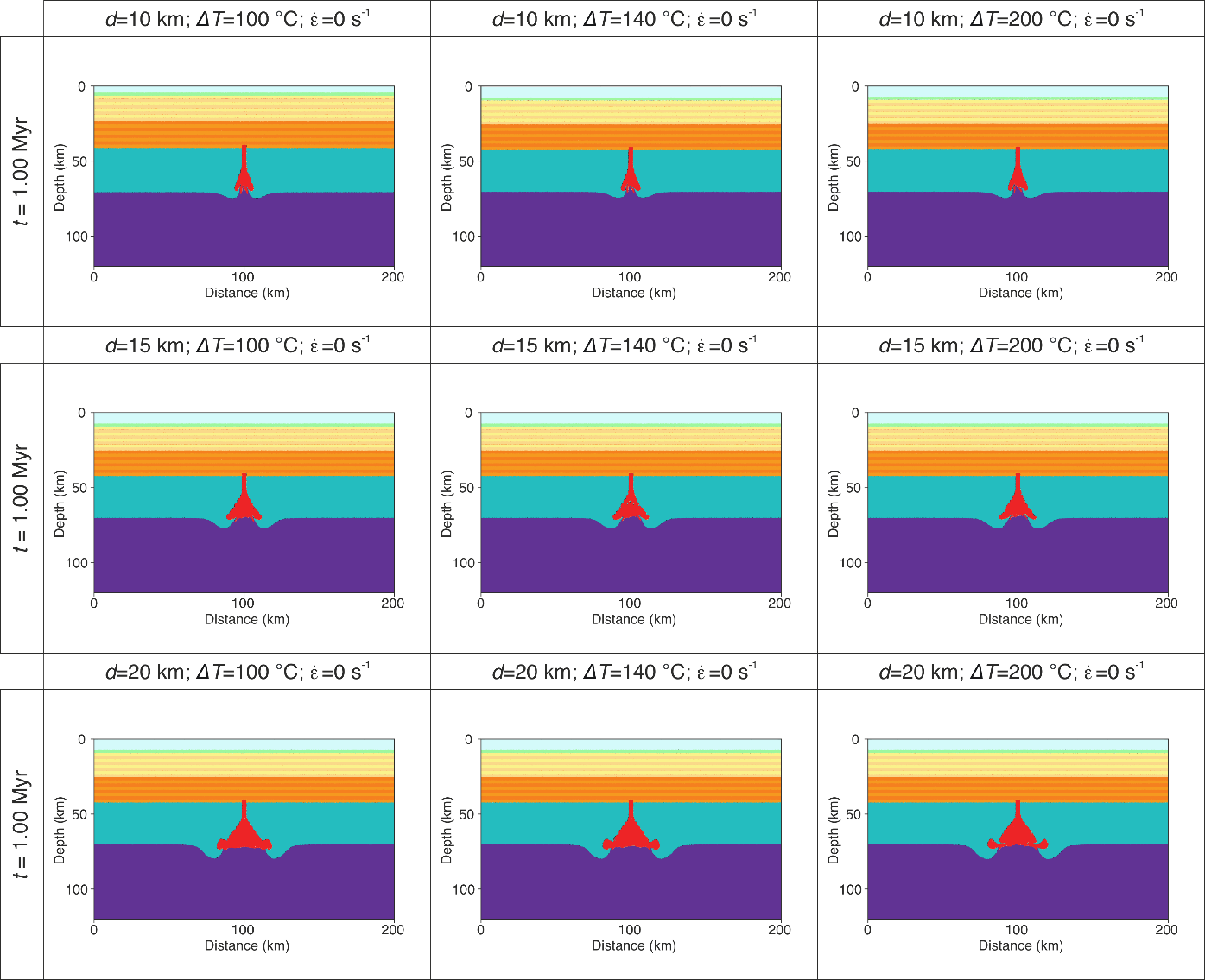 |
| --- |
| **Figure S4.** Evolution of rock composition after 1 Myr for simulations without background strain rate and involving a single hydrated mantle source with 10-20 km in diameter and *ΔT*=100-200 ºC. |

In supplementary Figure S5 we show modeling results as in supplementary Figure S4 but in terms of strain rate. Note that most of deformation is viscous and concentrated at the base of lithosphere. No plastic deformation is observed in the crust after 1 Myr of evolution.

| 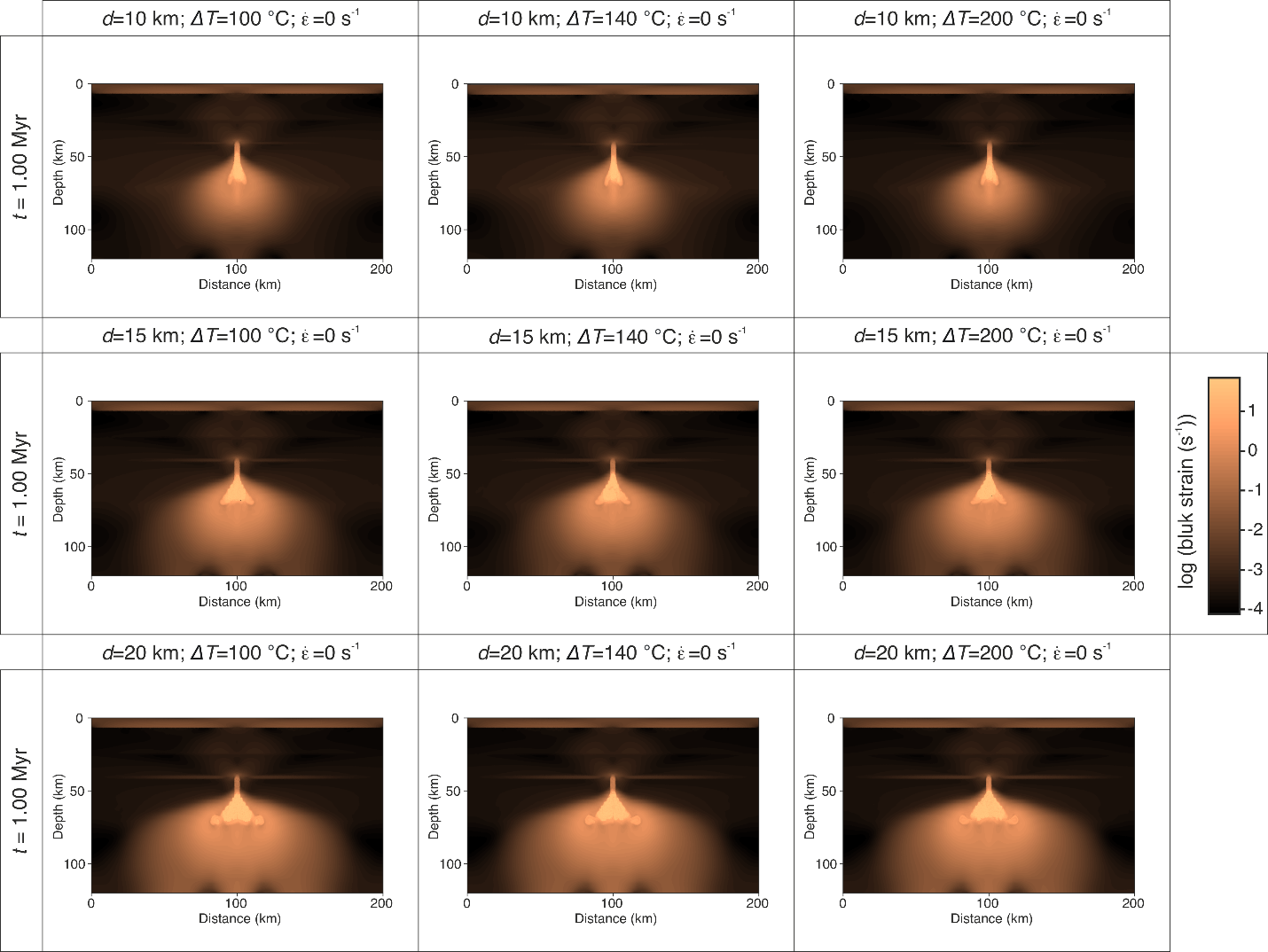 |
| --- |
| **Figure S5.** Evolution of strain rate after 1 Myr for simulations without extension and involving a single hydrated mantle source with 10-20 km in diameter and *ΔT*=100-200 ºC. |

Starting with supplementary Figure S6 we show modeling results for multiple thermal anomalies introduced at various time intervals in the simulation. In supplementary Figure S6 we present modeling results for simulations with two thermal anomalies (the 2^nd^ anomaly is inserted at different periods of time after model initiation (0.37-0.63 Myr)) of various sizes *d*=10-20 km composed of hydrated mantle. No background strain rates are applied to the modeling domain and the thermal anomaly includes a temperature excess range of *ΔT*=100-200 ºC. All numerical predictions are shown after 1 Myr of evolution and show no mantle transport through the continental crust.

| 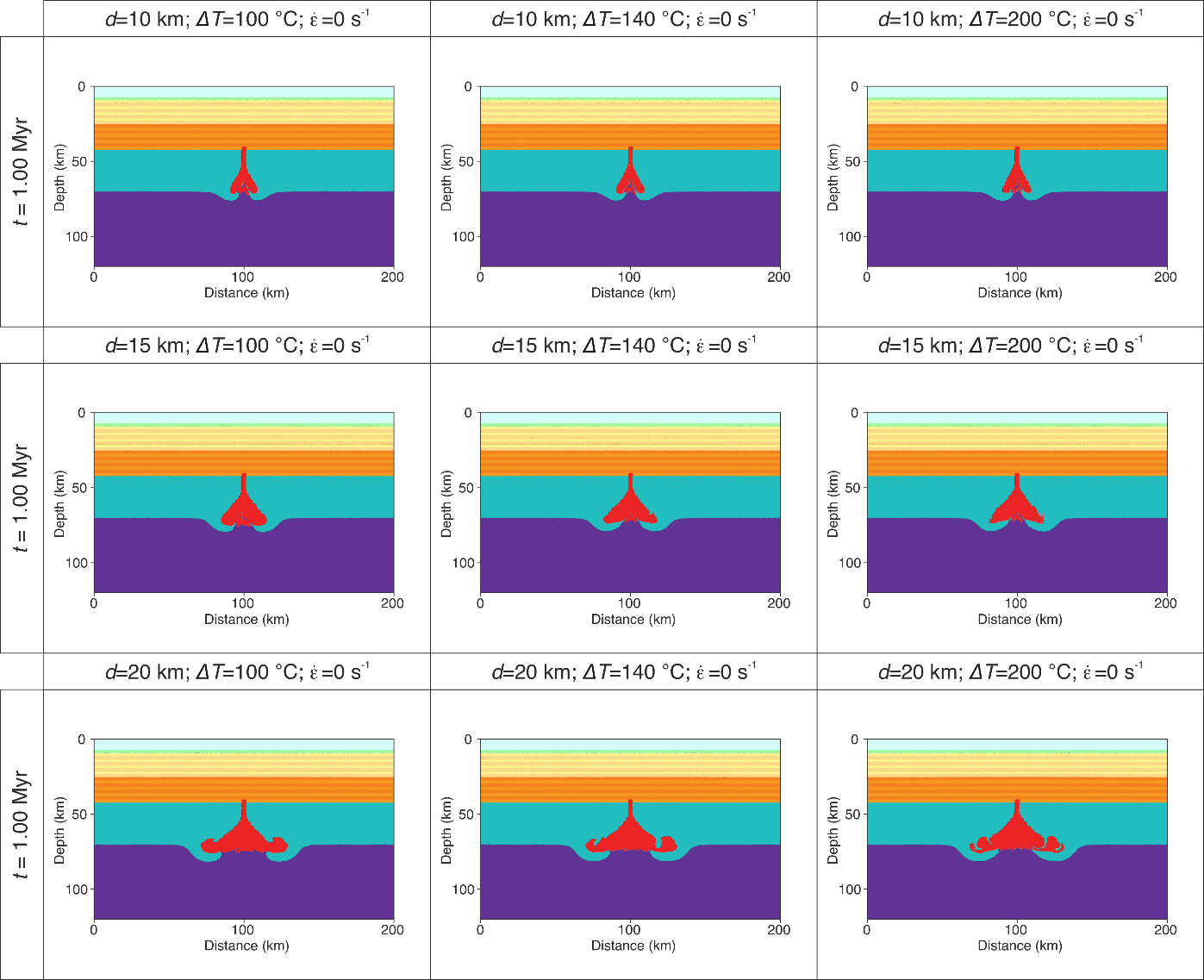 |
| --- |
| **Figure S6.** Evolution of rock composition after 1 Myr for simulations without background strain rate for several models involving a couple hydrated mantle source anomalies with 10-20 km in diameter and *ΔT*=100-200 ºC. |

In supplementary Figure S7 we present modeling results for simulations with three thermal anomalies (the 2^nd^ and the 3^rd^ anomalies are inserted at different periods of time after model initiation (~0.41-0.51 Myr)) of various sizes *d*=10-20 km and are composed of hydrated mantle. No background strain rates are applied to the modeling domain and the thermal anomaly includes a temperature excess range of *ΔT*=100-200 ºC. All numerical predictions are shown after 1 Myr of evolution and show no mantle transport through the continental crust, most of the deformation is viscously and laterally accommodated at the base of the lithosphere.

| 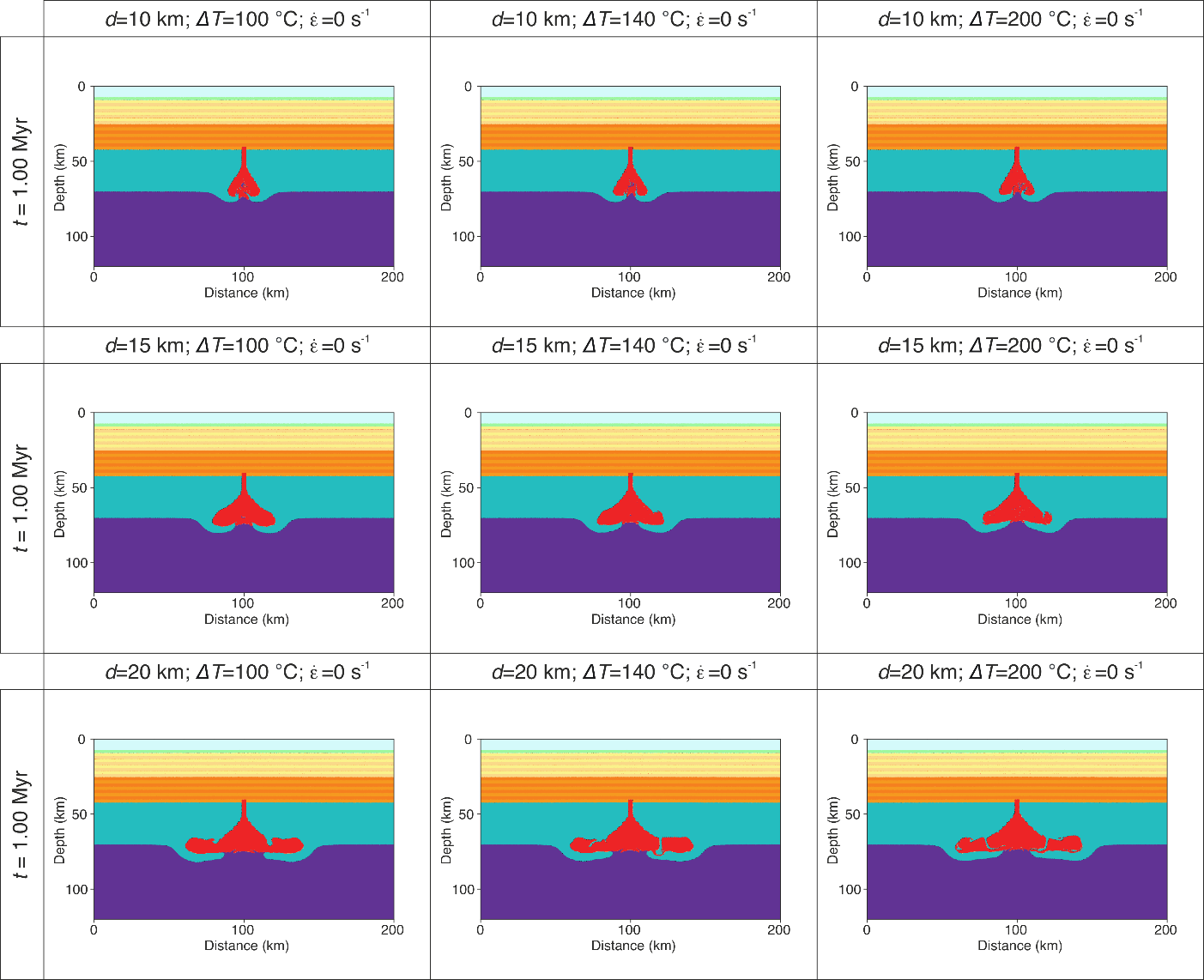 |
| --- |
| **Figure S7.** Evolution of rock composition after 1 Myr for simulations without background strain rate for several models involving three hydrated mantle source anomalies with 10-20 km in diameter and *ΔT*=100-200 ºC. |

In supplementary Figure S8 we present modeling results for simulations with three thermal anomalies (the 2^nd^ and the 3^rd^ anomalies are inserted at different periods of time after model initiation (~0.41-0.51 Myr)) of *d*=10 km in diameter and are composed of hydrated mantle. Background strain rates are applied to the modeling domain sides (4.756-7.927 x 10^-16^ s^-1^) and the thermal anomaly includes a temperature excess range of *ΔT*=100-200 ºC. All numerical predictions are shown after 1 Myr of evolution and show no mantle transport through the continental crust, most of the deformation is viscously and laterally accommodated at the base of the lithosphere.

| 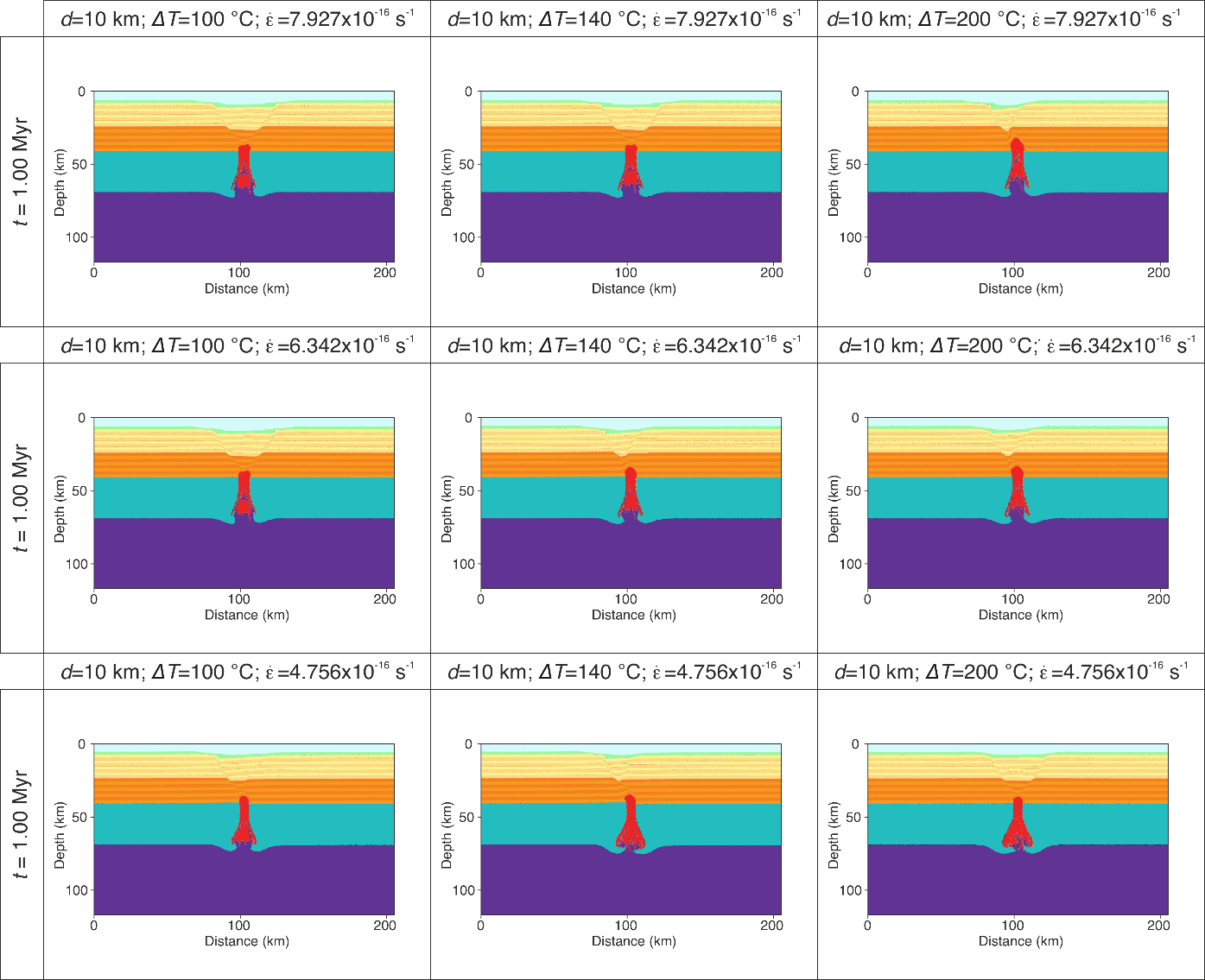 |
| --- |
| **Figure S8.** Evolution of rock composition after 1 Myr for simulations with background strain rates (4.756-7.927 x 10^-16^ s^-1^) for several models involving three hydrated mantle source anomalies with 10 km in diameter and *ΔT*=100-200 ºC. |

In supplementary Figure S9 we present modeling results for simulations with three thermal anomalies (the 2^nd^ and the 3^rd^ anomalies are inserted at different periods of time after model initiation (~0.41-0.51 Myr)) of *d*=15 km in diameter and are composed of hydrated mantle. Background strain rates are applied to the modeling domain sides (4.756-7.927 x 10^-16^ s^-1^) and the thermal anomaly includes a temperature excess range of *ΔT*=100-200 ºC. All numerical predictions are shown after 1 Myr of evolution and show no mantle transport through the continental crust, most of the deformation is viscously and laterally accommodated at the base of the lithosphere.

| 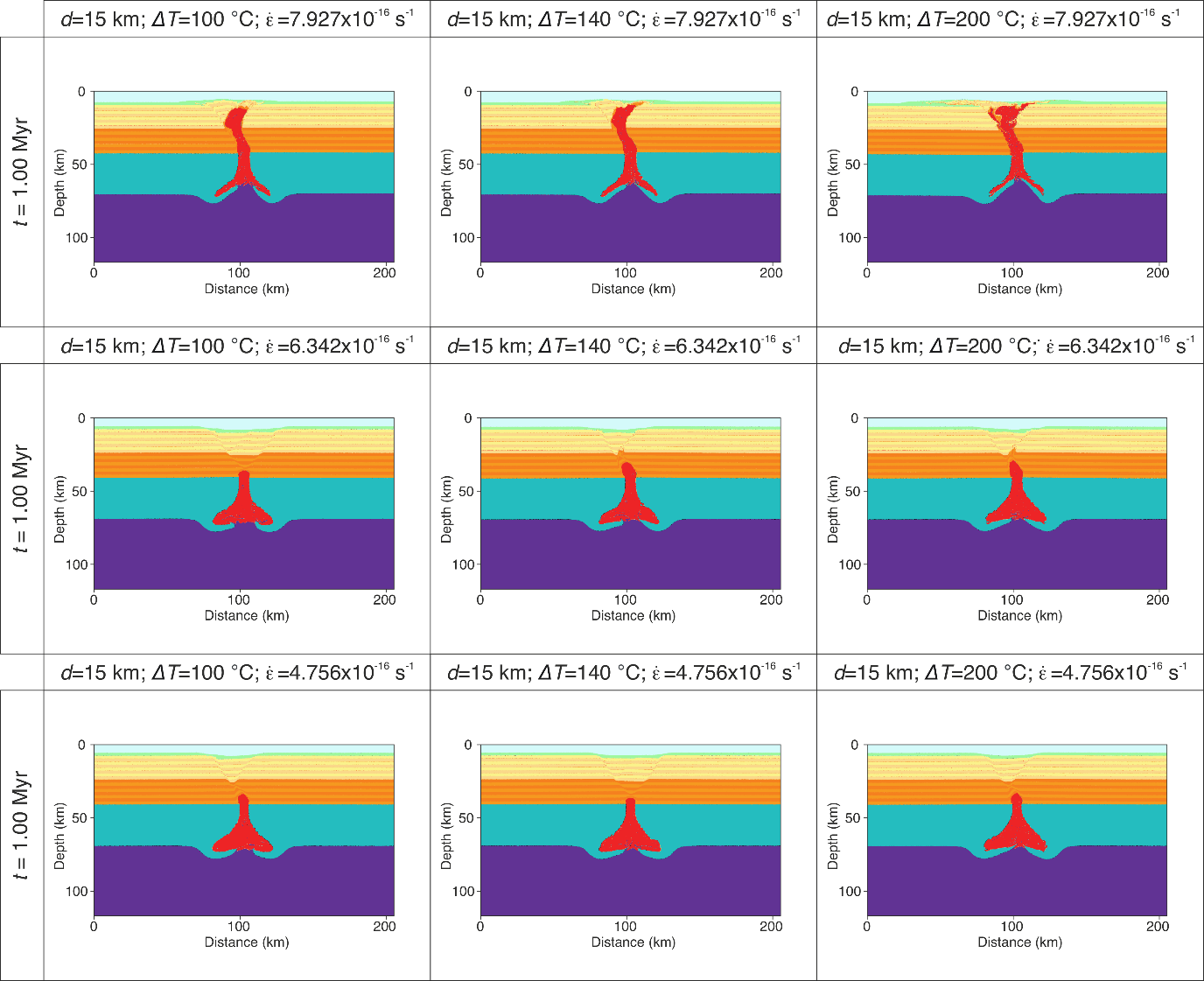 |
| --- |
| **Figure S9.** Evolution of rock composition after 1 Myr for simulations with background strain rates (4.756-7.927 x 10^-16^ s^-1^) for several models involving three hydrated mantle source anomalies with 15 km in diameter and *ΔT*=100-200 ºC. |

In supplementary Figure S10 we present modeling results for simulations with three thermal anomalies (the 2^nd^ and the 3^rd^ anomalies are inserted at different periods of time after model initiation (~0.41-0.51 Myr)) of *d*=20 km in diameter and are composed of hydrated mantle. Background strain rates are applied to the modeling domain sides (4.756-7.927 x 10^-16^ s^-1^) and the thermal anomaly includes a temperature excess range of *ΔT*=100-200 ºC. All numerical predictions are shown after 1 Myr of evolution and show no mantle transport through the continental crust, most of the deformation is viscously and laterally accommodated at the base of the lithosphere.

| 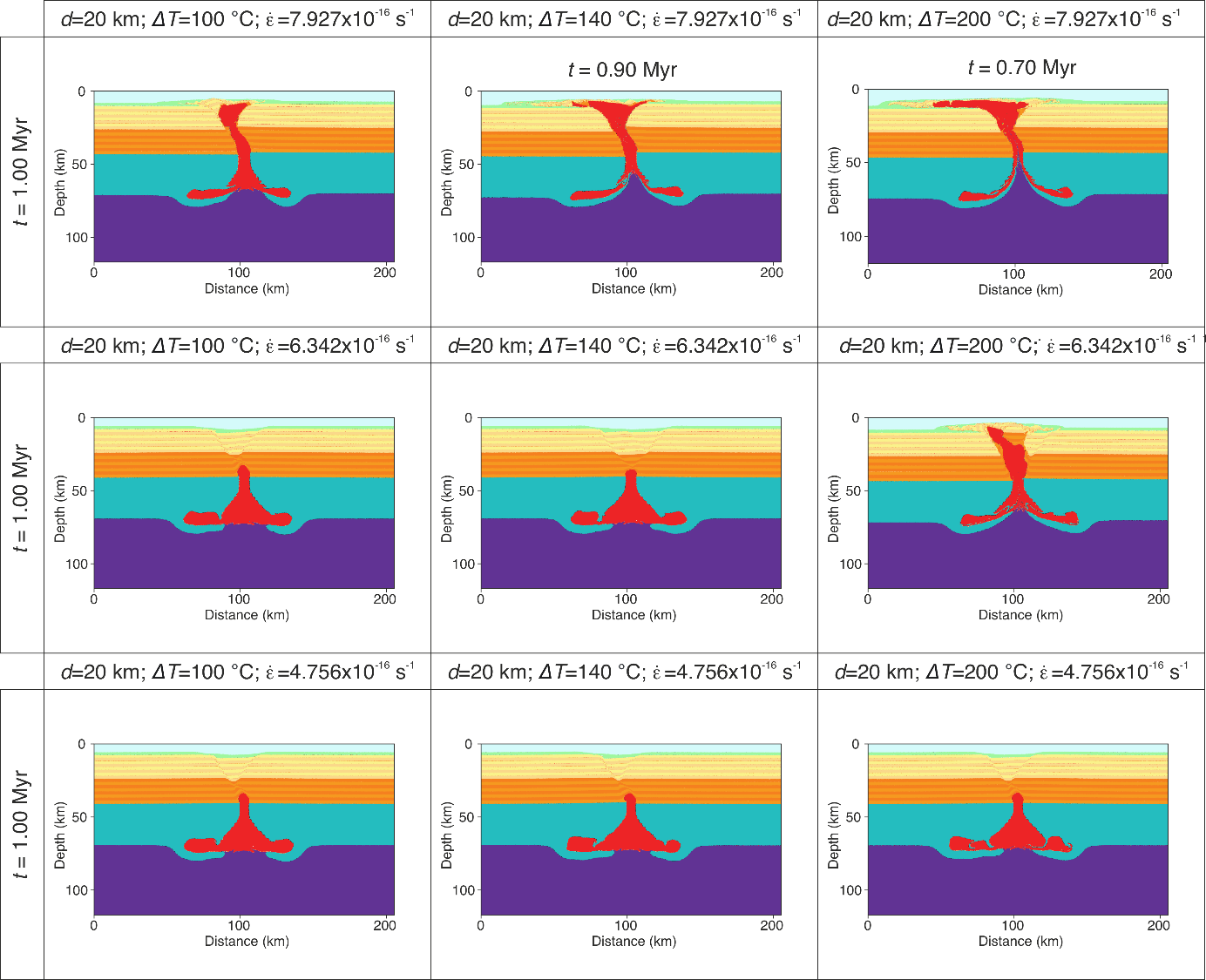 |
| --- |
| **Figure S10.** Evolution of rock composition after 1 Myr for simulations with background strain rates (4.756-7.927 x 10^-16^ s^-1^) for several models involving three hydrated mantle source anomalies with 20 km in diameter and *ΔT*=100-200 ºC. If simulation time is less than 1 Myr, this is indicated above the model domain. |

**3. Supplementary animations**

**Supplementary Movie 1.** Evolution of rock composition for 1 Myr for a simulation with background strain rates of 7.927 x 10^-16^ s^-1^ involving three hydrated mantle source anomalies with 15 km in diameter and *ΔT*=100 ºC introduced at various time intervals (0.44 Myr and 0.53 Myr) after the model initiation.

**Supplementary Movie 2.** Evolution of rock composition for 1 Myr for a simulation with background strain rates of 7.927 x 10^-16^ s^-1^ involving three hydrated mantle source anomalies with 15 km in diameter and *ΔT*=120 ºC introduced at various time intervals (0.42 Myr and 0.50 Myr) after the model initiation.

**Supplementary Movie 3.** Evolution of rock composition for 1 Myr for a simulation with background strain rates of 7.927 x 10^-16^ s^-1^ involving three hydrated mantle source anomalies with 20 km in diameter and *ΔT*=100 ºC introduced at various time intervals (0.43 Myr and 0.48 Myr) after the model initiation.

**4. Supplementary References**

Bittner, D., & Schmeling, H. (1995). Numerical modelling of melting processes and induced diapirism in the lower crust. *Geophysics*, *123*, 59–70.

Clauser, C., & Huenges, E. (1995). Thermal conductivity of rocks and minerals. Rock Physics and Phase Relations, AGU Reference Shelf 3, AGU, Washington, DC:, 105–126.

Galland, O. (2012). Experimental modelling of ground deformation associated with shallow magma intrusions. *Earth and Planetary Science Letters,* 317–318, 145–156. https://doi.org/10.1016/j.epsl.2011.10.017

Gerya, T. (2010). *Introduction to Numerical Geodynamic Modelling*. Cambridge University Press.

Gerya, T., & Yuen, D. A. (2007). Robust characteristics method for modelling multiphase visco-elasto-plastic thermo-mechanical problems. *Physics of the Earth and Planetary Interiors*, *163*, 83–105.

Hess, P. C. (1989) *Origin of Igneous Rocks*. Harvard University Press.

Hirschmann, M. M. (2000). Mantle solidus: Experimental constraints and the effects of peridotite composition. *Geochemistry, Geophysics, Geosystems*, **1**(10)**.** https://doi.org/10.1029/2000GC000070

Hofmeister, A. M. (1999). Mantle values of thermal conductivity and the geotherm from phonon lifetimes. *Science*, *283*, 1699–1706.

Holder, R. M., Viete, D. R., Brown, M., & Johnson, T. E. (2019). Metamorphism and the evolution of plate tectonics. *Nature*, *572*(7769), 378–381. https://doi.org/10.1038/s41586-019-1462-2

Johannes, W. (1985) *The significance of experimental studies for the formation of migmatites.* In

Ashworth, V. A. (ed.), Migmatites, Blackie, pp. 36–85.

Ranalli, G. (1995). Rheology of the Earth. In *Chapman and Hall* (Second).

Schmidt, M. W., & Poli, S. (1998). Experimentally based water budgets for dehydrating slabs and consequences for arc magma generation. *Earth Planet. Sci. Lett.*, *163*, 361–379.

Schmidt, M. W., & Poli, S. (2002). Petrology of subducted slabs. *Annu. Rev. Earth Science*, *30*, 207–235.

Turcotte, D., & Schubert, G. (2002). *Geodynamics* (Second). Cambridge University Press.

Turcotte D., & Schubert, G. (2014). *Geodynamics* (Third). Cambridge University Press.

van Wyk de Vries, B., Márquez, A., Herrera, R., Granja Bruña, J. L., Llanes, P., & Delcamp, A. (2014). Craters of elevation revisited: Forced-folds, bulging and uplift of volcanoes. *Bulletin of Volcanology*, **76**(11). https://doi.org/10.1007/s00445-014-0875-x
